# Restoration of Excitation‐Inhibition Balance and Improvement of Schizophrenia‐like Behavioral Deficits via Electroacupuncture

**DOI:** 10.1002/cns.70971

**Published:** 2026-06-10

**Authors:** Chaofan Wan, Yucen Xia, Jinglan Yan, Weipeng Lin, Yuanjia Zheng, Yiqiu Lin, Lin Yao, Yongjun Chen

**Affiliations:** ^1^ School of Chinese Medicine, Guangdong Provincial Engineering and Technology Research Center of Light and Health Guangdong Pharmaceutical University Guangzhou China; ^2^ South China Research Center for Acupuncture and Moxibustion, Medical College of Acu‐Moxi and Rehabilitation Guangzhou University of Chinese Medicine Guangzhou China; ^3^ Institute of Acupuncture and Moxibustion Shandong University of Traditional Chinese Medicine Jinan China; ^4^ Department of Rehabilitation Medicine The Third Affiliated Hospital of Sun Yat‐Sen University Guangzhou China; ^5^ Shandong Key Laboratory of Innovation and Application Research in Basic Theory of Traditional Chinese Medicine Shandong University of Traditional Chinese Medicine Jinan China; ^6^ Key Laboratory of Traditional Chinese Medicine Classical Theory, Ministry of Education Shandong University of Traditional Chinese Medicine Jinan China; ^7^ Institute of Traditional Chinese Medicine and Neuroscience Shandong University of Traditional Chinese Medicine Jinan China

**Keywords:** electroacupuncture, excitation‐inhibition balance, hippocampus, schizophrenia

## Abstract

**Background:**

Imbalances between excitatory and inhibitory signals have been implicated in the pathophysiology of schizophrenia. Electroacupuncture (EA) is commonly used in clinical practice to address neuropsychiatric conditions. However, the exact therapeutic mechanisms underlying EA's effects on schizophrenia remain poorly understood.

**Methods:**

We employed Erbb4‐nNos^−/−^ mice, which model the genetic deficits observed in schizophrenia, as a model system for our study. The locomotor activity, social behavior, sensorimotor gating ability, working memory, spatial learning and memory, and motor skills and coordination of mice were assessed using the open‐field test, three‐chamber social interaction test, prepulse inhibition (PPI) test, Y‐maze, Morris water maze, and rotarod tests. Whole‐cell patch‐clamp recordings were performed to detect excitatory and inhibitory synaptic transmission and the intrinsic excitability of pyramidal neurons.

**Results:**

EA alleviated behavioral deficits associated with schizophrenia in these mice. Notably, EA also enhanced GABAergic transmission and decreased the excitability of pyramidal neurons. Simultaneously, the selective nNOS inhibitor L‐NPA blocked the therapeutic effects of EA on behavioral deficits and synaptic transmission.

**Conclusions:**

EA improved schizophrenia‐like behavioral impairments and restored the balance between excitatory and inhibitory signaling, possibly through the upregulation of presynaptic GABA release. Furthermore, nitric oxide (NO) signaling may play a critical role in the observed benefits. These findings underscore the significance of excitatory–inhibitory (E/I) balance in mediating the therapeutic effects of EA, highlighting its potential as an intervention strategy for schizophrenia.

## Graphical abstract

Ablation of nNos from Erbb4‐positive neurons induces impaired GABAergic transmission and disruption of excitatory and inhibitory balance, which further cause hyperactivity of pyramidal neurons, resulting in schizophrenia‐relevant behavioral deficits. EA treatment rescues decreased GABAergic transmission and restores excitatory and inhibitory balance by the endogenous nNOS system, which contributes to the improvement of behavioral deficits.

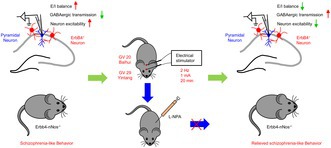

## 1 Introduction

Schizophrenia is a debilitating, chronic psychiatric disorder that disrupts cognition, perception, emotional regulation, and social functioning [1, 2]. Affecting roughly 1% of the global population, schizophrenia typically manifests in late adolescence or early adulthood, creating significant challenges for individuals, families, and healthcare systems worldwide [3]. The disorder presents with a range of symptoms: positive symptoms include hallucinations, delusions, and disorganized speech or behavior, while negative symptoms include social withdrawal and anhedonia, and blunted emotional expression. Cognitive deficits, such as impairments in attention, memory, and executive function, are also prominent and often persist throughout the course of the illness, contributing to long‐term functional impairment [2]. Neurobiological research has revealed several abnormalities in neurotransmitter systems in schizophrenia, with numerous studies highlighting disruptions in dopaminergic, serotonergic, glutamatergic, and GABAergic signaling [4]. Despite advances in pharmacological treatments based on various hypotheses, current therapies, particularly antipsychotic medications, have limited efficacy for negative and cognitive symptoms, and the intervention of alternative treatments may be required [5].

Electroacupuncture (EA), a modified form of acupuncture that applies controlled electrical stimulation to specific acupoints, offers a more consistent and controllable alternative for treatment [6, 7]. Previous studies have indicated that EA is conducive to improving cognitive impairment in Alzheimer's disease [8], vascular cognitive impairment [9], and depression [10]. This makes EA a promising candidate for treating neuropsychiatric disorders, including schizophrenia, where it has recently garnered attention [11, 12]. Our recent study demonstrated that prenatal EA modulates maternal‐fetal immune equilibrium to mitigate offspring neurodevelopmental impairments including hyperactivity, impaired cognitive function, and sensorymotor gating by a brain‐to‐splenic signal [11]. Furthermore, EA mitigates the weight gain and lipid metabolism disturbances induced by the antipsychotic drug Olanzapine via a liver‐brain regulatory mechanism, which may improve treatment adherence in schizophrenia patients [12]. This evidence indicates that EA has potential clinical value in the treatment of schizophrenia. Although limited preclinical studies have shown that EA can modulate neurotransmitter levels and alleviate abnormal behaviors in schizophrenia models, whether EA can exert effects by regulating synaptic transmission and E/I balance in the hippocampus remains to be verified.

Although the precise causes of schizophrenia are not fully understood, it is widely regarded as a multifactorial condition resulting from intricate interactions between genetic and environmental factors [13]. ErbB4, a receptor tyrosine kinase, is widely expressed in GABAergic neurons throughout the cortex, hippocampus, and amygdala [14]. Genetic variations in Erbb4 may elevate schizophrenia risk by disrupting neural circuit formation and function [15, 16]. Neuronal nitric oxide synthase (nNos), also referred to as Nos1, plays a critical role in the central nervous system. It modulates synaptic plasticity and neurotransmitter release through nitric oxide (NO) signaling [17]. Polymorphisms in nNos may impair brain signaling pathways and thereby contributing to the pathogenesis of schizophrenia [18, 19]. Our previous study demonstrated that most Erbb4‐positive (Erbb4^+^) neurons co‐expressed nNOS in the mouse hippocampus, and activation of ErbB4 by neuregulin 1 (NRG1) upregulated phosphorylation of nNOS and increased GABAergic synaptic transmission [19]. In addition, mice with a genetic deletion of nNos in Erbb4^+^ neurons (Erbb4‐nNos^−/−^) exhibit multiple schizophrenia‐related behavioral deficits and impaired E/I balance [19].

In the present study, we used Erbb4‐nNos^−/−^ mice, which replicate the genetic deficits seen in schizophrenia, to investigate the therapeutic mechanisms of EA. As anticipated, EA treatment improved several schizophrenia‐related behavioral deficits, including hyperactivity, impaired sensorimotor gating, and deficits in working memory. Critically, EA restored GABAergic transmission and re‐established E/I balance, potentially by upregulating presynaptic GABA release. Furthermore, both application of the nitric oxide synthase (NOS) inhibitor L‐NAME and the selective nNOS inhibitor L‐NPA blocked the therapeutic effects of EA on these behavioral deficits. Taken together, our findings provide mechanistic insights into the therapeutic effects of EA in a schizophrenia mouse model.

## 2 Materials and Methods

### 2.1 Animals

The Erbb4^CreER/+^ mice (JAX# 012360), with tamoxifen‐inducible Cre under the control of the Erbb4 gene, were obtained from Jackson Laboratory, while Floxed nNos (nNos^f/f^) mice were kindly donated by Dr. Jennifer S. Pollock [20]. To achieve nNOS deletion specifically in Erbb4‐expressing neurons, nNos^f/f^ mice were mated with Erbb4^CreER/+^ mice, resulting in the generation of Erbb4^CreER/+^; nNos^f/f^ (Erbb4‐nNos^−/−^) mice, with nNos^f/f^ littermates used as controls [19]. Mice were maintained on a 12 h light/dark cycle (lights on at 7:00) with ad libitum food and water. Behavioral testing and patch‐clamp experiments were performed on 13‐ to 16‐week‐old males.

### 2.2 Drug Administration

As previously described [19], eight‐week‐old mice were injected daily with 100 mg/kg tamoxifen (T5648, Sigma‐Aldrich, St. Louis, MO, USA) for five consecutive days. L‐NAME (N5751, Sigma‐Aldrich, St. Louis, MO, USA) or L‐NPA (80,587, Cayman Chemical, Ann Arbor, MI, USA) was administered intraperitoneally to the mice half an hour prior to the initiation of behavioral studies at doses of 30 mg/kg and 20 mg/kg, respectively.

### 2.3 Electroacupuncture Treatment

For the treatment, mice received acupuncture (AC), electroacupuncture (EA), and sham EA or intervention under light anesthesia. Anesthesia was induced using 3% isoflurane and maintained with 2% isoflurane using a mask [21]. Two acupuncture needles (0.16 × 7 mm, Hwato Appliance Factory, China) were gently inserted 2–3 mm deep into the acupoints of GV20 (Baihui) and GV29 (Yintang) in mice [22]. EA stimulation was performed with an intensity of 1 mA and a frequency of 2 Hz for 20 min once every other day for 20 days by using a Master‐8 Eight Channel Programmable Pulse Generator (AMPI) and 2 ISO‐Flex stimulus isolators (AMPI). AC intervention was administered without electrical stimulation, while sham EA was applied following the same protocol but with a nonelectrical wooden toothpick instead of a stainless‐steel needle [23].

### 2.4 Slice Preparation

Hippocampal slices were prepared as described before. In brief, mice were first sacrificed with pentobarbital (100 mg/kg i.p.) and transcardially perfused with ice‐cold oxygenated (95% O_2_ and 5% CO_2_) cutting solution before decapitation. Subsequently, brains were promptly extracted and kept in ice‐cold oxygenated cutting solution. The cutting solution contained the following (in mM): 220 sucrose, 26 NaHCO_3_, 2.5 MgSO_4_, 2.5 KCl, 1.3 CaCl_2_‐2H_2_O, 1 NaH_2_PO_4_‐2H_2_O, and 10 D‐glucose. Coronal hippocampal slices (300 μm) were precisely prepared by a VT1200S vibratome (Leica, Germany) in ice‐cold cutting solution. The slices were then incubated in oxygenated regular artificial cerebrospinal fluid (ACSF) including (in mM): 126 NaCl, 26 NaHCO_3_, 3 KCl, 1.2 NaH_2_PO_4_‐2H_2_O, 1 MgSO_4_, 2.0 CaCl_2_‐2H_2_O, and 10 D‐glucose for 30 min at 33°C and 1 h at room temperature (25°C ± 1°C) before recording.

### 2.5 Whole‐Cell Patch Clamp Recordings

To conduct electrophysiological recordings [19], hippocampal slices were placed in the recording chamber with continuous perfusion of oxygenated regular ACSF (3 mL/min) at 32°C–34°C. Whole‐cell patch‐clamp recordings from CA1 neurons were visualized with infrared optics using an upright microscope (Eclipse FN1, Nikon, Japan) equipped with a Digital CMOS camera (C11440‐42 U, Hamamatsu, Japan). The CA1 pyramidal neurons in the hippocampus were identified based on their location and shape. The patch pipettes (4–6 MΩ) were pulled from borosilicate glass with filament (BF150‐86‐10, Sutter Instruments, USA) by a micropipette puller (P‐97, Sutter Instruments, USA).

For recording spontaneous excitatory postsynaptic currents and inhibitory postsynaptic currents (sEPSCs/sIPSCs), patch pipettes were filled with a solution containing (in mM): 125 CsCH_3_SO_3_, 5 CsCl, 10 phosphocreatine, 10 HEPES, 0.2 EGTA, 1 MgCl2, 4 Mg‐ATP, 0.3 Na‐GTP, and 5 QX‐314 (pH 7.30, 280 mOsm). The voltage clamp recordings were performed at −60 mV for sEPSC recordings and at +10 mV for sIPSC recordings.

To record miniature excitatory postsynaptic currents (mEPSCs), 1 μM tetrodotoxin (TTX) and 20 μM BMI were added to ACSF before recording. Pipettes were filled with a K^+^‐based solution including (in mM) 105 K‐gluconate, 30 KCl, 10 HEPES, 10 phosphocreatine, 4 ATP‐Mg, 0.3 GTP‐Na, and 0.3 EGTA (pH 7.35, 285 mOsm). The K^+^‐based intracellular solution was also used to record action potentials (APs). To characterize membrane and firing properties of neurons, we applied hyperpolarizing and depolarizing current steps (−200 to 350 pA) for 500 ms at 0.2 Hz in current‐clamp configuration.

Both mIPSCs and eIPSCs were pharmacologically isolated in the presence of DL‐AP5 (100 μM) and CNQX (20 μM) at a holding potential of −70 mV and were confirmed by the addition of 20 μM bicuculline methiodide (BMI). For miniature IPSCs (mIPSCs) recording, we added 1 μM TTX to ACSF. Pipettes were filled with internal solution (in mM): 140 CsCl, 10 HEPES, 0.2 EGTA, 1 MgCl_2_, 4 ATP‐Mg, 0.3 GTP‐Na, and 5 QX‐314 (pH 7.25, 280 mOsm). To record evoked inhibitory postsynaptic currents (eIPSCs), we stimulated the axons in the stratum radiatum with a two‐concentric bipolar stimulating electrode (CBARC75, FHC, USA) connected to a stimulus isolation unit (ISO‐Flex Stimulus Isolator; AMPI) at a frequency of 0.033 Hz. For the pipette solution, the concentration of CsCl was decreased to 60 mM and 100 mM CsCH_3_SO_3_ was added. To measure the paired‐pulse ratio (PPR) of eIPSCs, four continuous pulses were applied at an interval of 50 ms.

Data were collected by the Multiclamp 700B amplifier and 1550A digitizer (Molecular Devices, USA), digitized at 10 kHz and filtered at 1 kHz. We collected data when the series resistance fluctuated within 20% of initial values and analyzed it using pClamp 10.7 software (Molecular Devices, USA).

### 2.6 Behavioral Assay

Behavioral tests were performed as previously described [19]. Before tests, mice were placed in the testing room for at least 1 h for habituation. The apparatus and areas were routinely cleaned with 75% ethanol and dried thoroughly after each test session.

### 2.7 Open Field Test

Mice were gently placed in the center of a cubic plastic box (40 × 40 × 40 cm) and allowed to explore for 30 min. The mouse movements were recorded with an infrared camera placed above the box. The total distance, time in the center area, and central distance during 30 min were measured (Jiliang Software Technology, Shanghai, China).

### 2.8 Social Interaction Test

For the social interaction test, a blue rectangular Plexiglas arena (60 × 40 × 30 cm) was divided into three equal interconnected compartments. Each lateral compartment contained a clear cylinder fixed to the floor. One cylinder later served as the social stimulus enclosure, the other remained empty. Testing proceeded in three consecutive phases: (1) Habituation: mice were placed in the central compartment and allowed 5 min of free exploration of the arena without cylinders. (2) Object acclimation: two empty cylinders were positioned in the lateral compartments and mice explored for 10 min. (3) Sociability: an age‐matched, unfamiliar adult male C57BL/6J mouse was introduced into one cylinder (social side), the opposite cylinder was empty (nonsocial side), and the test mouse explored for 10 min. Each session was recorded, and the time spent around each cylinder was analyzed using the tracking system (Jiliang Software Technology, Shanghai, China).

### 2.9 Prepulse Inhibition (PPI)

Experiments were performed in ventilated startle chambers (San Diego Instruments, San Diego, CA, USA). After a 5‐min acclimation period with continuous 70‐dB broadband background noise, each mouse received a pseudorandom sequence of 24 trials: 12 pulse‐alone trials consisting of a 120 dB, 20 ms broadband burst, and 12 prepulse/pulse trials in which a 20 ms prepulse (75, 80, or 85 dB) preceded the 120 dB pulse by 100 ms (onset‐to‐onset). Startle responses were detected by a piezoelectric transducer and peak amplitude (arbitrary units) was recorded. PPI (%) for each prepulse intensity was computed as follows: 100 × (startle amplitude for pulse alone − startle amplitude for pulse with prepulse)/startle amplitude for pulse alone.

### 2.10 Morris Water Maze

A circular pool (120 cm in diameter) molded from matte‐blue polypropylene was enclosed by white curtains extending from the rim to the ceiling to eliminate extra‐maze cues. The pool was filled with water (20°C–22°C) rendered opaque by the addition of non‐toxic white tempera paint. A transparent Plexiglas escape platform (10 cm diameter) was positioned 1 cm beneath the surface in the center of the designated target quadrant. Spatial acquisition (days 1–4): each mouse received 4 training trials per day. Release points were pseudo‐randomized across four cardinal compass positions; the hidden platform remained in a fixed location. Trials terminated when the mouse climbed onto the platform or after 60 s, whichever occurred first. Latency to escape was recorded. Probe test (late day 4): the platform was removed and mice were released from the quadrant opposite the former target. A 60‐s probe trial was conducted, and the mice were scored for the number of platform crossings. Visible‐platform cue test (day 5): the platform was raised 1 cm above the surface and marked with a striped flag. Mice were assessed for their ability to locate a visible platform within 60 s, with any mouse experiencing two 60‐s trials being eliminated from the study. Escape latency and the number of platform crossings were analyzed using the tracking system (Jiliang Software Technology, Shanghai, China).

### 2.11 Y‐Maze

A symmetrical Y‐shaped arena consisted of three identical arms (25 × 10 × 25 cm) intersecting at 120° angles and labeled A, B, and C for reference. Each mouse was introduced at the distal end of arm A and allowed to explore freely for a single 5‐min session. Limb positioning within an arm was considered an arm entry. An alternation was defined as the consecutive entry into all three arms without repetition. The mouse activity and spontaneous behavioral alternations were recorded by the tracking system (Jiliang Software Technology, Shanghai, China). The percentage of spontaneous alternation was calculated as follows: % alternation = ([number of alternations] / [total number of arm entries −2]) × 100.

### 2.12 Rotarod Test

Motor coordination and equilibrium were assessed in a motor‐driven rotarod apparatus (Jiliang Software Technology, Shanghai, China). The rod accelerated uniformly from 4 rpm to 40 rpm over 300 s. Each mouse was placed on the stationary bar and rotation commenced immediately upon software trigger. Trials ended when the animal fell, triggering the infrared detection beam, or after 300 s had elapsed. Mice received two trials per day on two consecutive days (inter‐trial interval of 30 min). Latency to fall (s) from the rod was recorded automatically by the recording system (Jiliang Software Technology, Shanghai, China).

### 2.13 Statistical Analysis

Statistical analyses were conducted using SPSS version 21.0 (SPSS Inc., Chicago, IL, USA) or GraphPad Prism 7 (GraphPad Software Inc., San Diego, CA, USA). The presentation of all data adhered to the format of means ± SEM. An unpaired *t*‐test was used for comparisons between two groups, whereas one‐way or two‐way ANOVA was applied for comparisons among three or four groups. The number of animals, recorded neurons, or independent experiments is provided in figure legends. All tests were two‐sided, and statistical significance was considered at *p* < 0.05.

## 3 Results

### 3.1 EA Improves Schizophrenia‐Related Behavioral Deficits in Erbb4‐nNos ^−/−^ Mice

**FIGURE 1:**
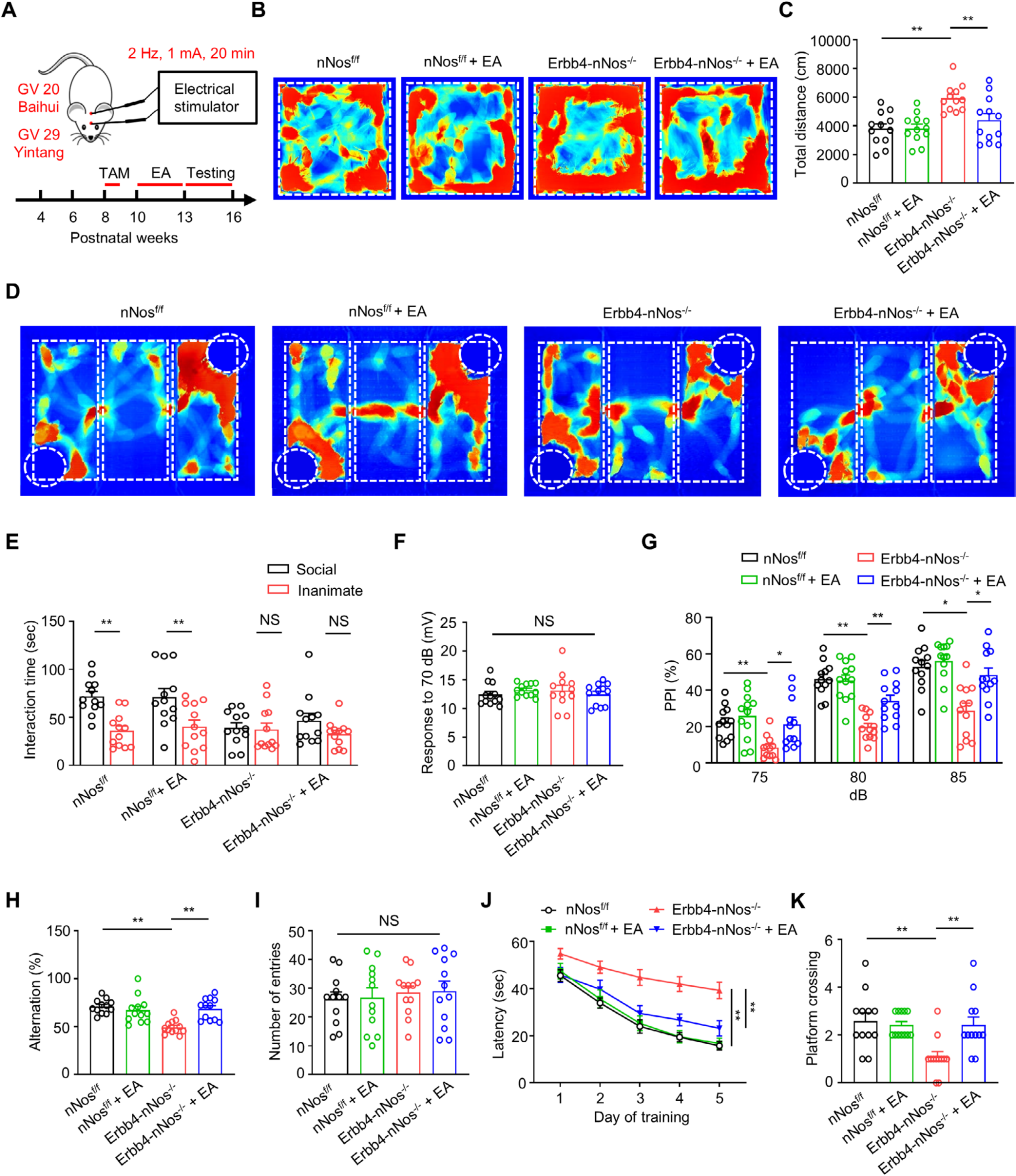
EA improves schizophrenia‐relevant behavioral deficits in Erbb4‐nNos^−/−^ mice. **(A)** Scheme of experimental design. Top: The schematic diagram of EA treatment of GV20 (Baihui) and GV29 (Yintang) acupoints in mice at an intensity of 1 mA/2 Hz for 20 min; Bottom: Experimental design and timeline of Tamoxifen injection, EA treatment and behavioral experiment. **(B‐C)** Representative activity tracking **(B)** and total distance **(C)** from the different groups, including nNos^f/f^, nNos^f/f^ + EA, Erbb4‐nNos^−/−^ and Erbb4‐nNos^−/−^ + EA groups, in open field test. **(C)** One‐way ANOVA with Bonferroni's multiple comparisons test, *N* = 12 per group; *F* = 8.385, **p < 0.01. **(D‐E)** Representative activity tracking **(D)** and interaction time **(E)** in the three‐chamber social test. **(E)** Two‐way ANOVA with Bonferroni's multiple comparisons test, *N* = 12 per group; *F* = 4.158, **p < 0.01. **(F‐G)** Quantification of response to 70 dB **(F)** and percentage of PPI **(G)** in prepulse inhibition test. **(F)** One‐way ANOVA with Bonferroni's multiple comparisons test, *N* = 12 per group; *F* = 0.4831, *p* = 0.6957. NS, not significant. **(G)** Two‐way Repeated Measures ANOVA with Bonferroni's multiple comparisons test, *N* = 12 per group; *F* = 19.63, *p < 0.05, **p < 0.01. **(H‐I)** Percentage of spontaneous alternation **(H)** and the number of arm entries **(I)** in Y‐maze. **(H)** One‐way ANOVA with Bonferroni's multiple comparisons test, *N* = 12 per group; *F* = 11.15, **p < 0.01. **(I)** One‐way ANOVA with Bonferroni's multiple comparisons test, *N* = 12 per group; *F* = 0.2003, *p* = 0.8956. NS, not significant. **(J‐K)** Quantification of latency **(J)** and platform crossing **(K)** in the water maze. **(J)** Two‐way Repeated Measures ANOVA with Bonferroni's multiple comparisons test, *N* = 12 per group; *F* = 26.79, **p < 0.01. **(K)** One‐way ANOVA with Bonferroni's multiple comparisons test, *N* = 12 per group; *F* = 6.501, **p < 0.01. Data are mean ± SEM. *N* indicates the number of biologically independent samples, mice per group.

Pharmacological models are commonly used in preclinical schizophrenia research because they effectively replicate the behavioral phenotypes associated with the disorder. However, schizophrenia is a complex mental illness with significant genetic underpinnings, making the development of genetically based animal models particularly valuable for research. In our previous study, we demonstrated that conditional knockout of nNos in Erbb4‐positive neurons in adult male mice induced schizophrenia‐like behaviors, which could be reversed with clozapine, a second‐generation antipsychotic used to treat schizophrenia [19]. In this study, we sought to investigate the therapeutic mechanisms of EA in treating schizophrenia using this genetic model (Figure S1A). For EA treatment, we targeted the GV20 (Baihui) and GV29 (Yintang) acupoints in mice, applying electrical stimulation at an intensity of 1 mA/2 Hz for 20 min every other day over the course of 20 days (Figure 1A). A battery of behavioral tests was performed to assess the effects of EA on schizophrenia‐related deficits. In the open‐field test, Erbb4‐nNos^−/−^ mice exhibited increased locomotor activity, reflected by a longer total distance traveled compared to control mice. This hyperactivity phenotype, characteristic of schizophrenia, was significantly reversed by EA treatment (Figure 1B,C). However, no significant differences were observed between groups in the amount of time spent in the center of the arena (Figure S1B). In the three‐chamber social interaction test, both Erbb4‐nNos^−/−^ mice with and without EA treatment spent less time interacting with a stimulus mouse compared to control mice, indicating deficits in social behavior (Figure 1D,E). The prepulse inhibition (PPI) test, which measures sensorimotor gating commonly disrupted in schizophrenia [24], revealed a lower level of PPI in Erbb4‐nNos^−/−^ mice. However, EA treatment increased PPI, suggesting that EA ameliorates sensorimotor gating impairments in these mice (Figure 1F,G). In the Y‐maze, used to assess working memory, the Erbb4‐nNos^−/−^ + EA group exhibited a significantly higher percentage of alternations compared to the Erbb4‐nNos^−/−^ group (Figure 1H), with a similar number of arm entries between the groups (Figure 1I). This indicates that EA improves working memory in Erbb4‐nNos^−/−^ mice. In the water maze, the Erbb4‐nNos^−/−^ + EA group showed reduced latency to locate the hidden platform and a higher frequency of platform crossings during the testing period, suggesting enhanced spatial memory (Figure 1J,K). Importantly, the rotarod test, which evaluates motor skills and coordination, revealed no differences across the groups, indicating that EA did not affect motor performance (Figure S1C). Additionally, to further verify the specificity of EA effects and clarify the independent role of acupuncture, we performed sham EA and conventional acupuncture (AC) in knockout mice and observed the potential effects of these interventions on animal behaviors. The results showed that sham EA failed to improve abnormal behaviors in the mice, whereas AC exerted a moderate ameliorative effect (Figure S2). Overall, these results demonstrate that EA treatment significantly improves several behavioral deficits relevant to schizophrenia in Erbb4‐nNos^−/−^ mice.

### 3.2 EA Restores Hippocampal E/I Balance in Erbb4‐nNos ^−/−^ Mice

**FIGURE 2:**
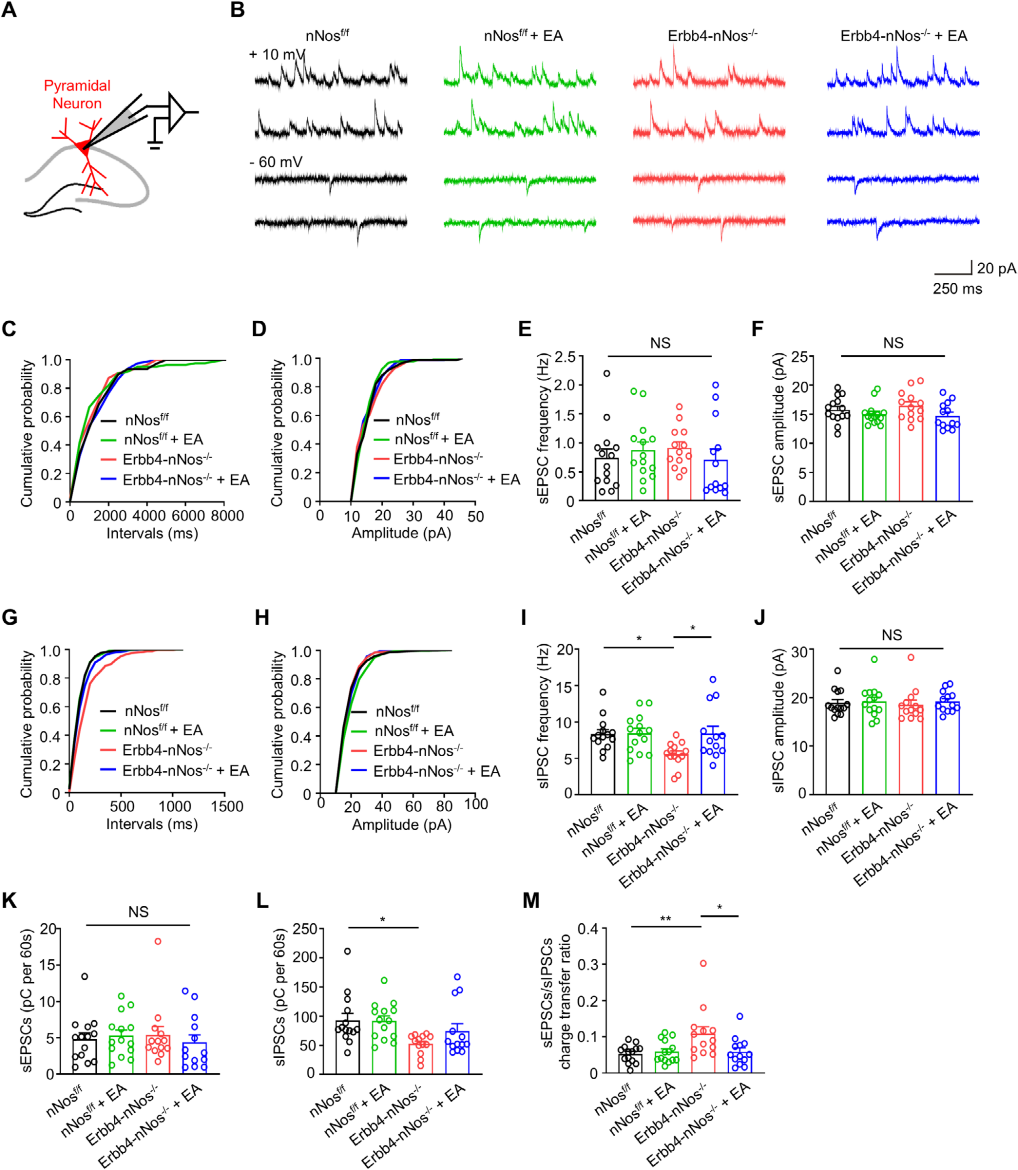
EA restores hippocampal E/I balance in Erbb4‐nNos^−/−^ mice. **(A)** Schematic representation of whole‐cell recordings from pyramidal neurons in hippocampus. **(B)** Representative traces of sEPSCs (−60 mV) and sIPSCs (+ 10 mV) in CA1 pyramidal neurons in the different groups, including nNos^f/f^, nNos^f/f^ + EA, Erbb4‐nNos^−/−^ and Erbb4‐nNos^−/−^ + EA groups. Scale bar = 250 ms, 20 pA. **(C‐D)** Cumulative plots of sEPSC interevent intervals **(C)** and amplitude **(D)**. **(E‐F)** Quantification of mean values of sEPSC frequency **(E)** and amplitude **(F)**. **(E)** One‐way ANOVA with Bonferroni's multiple comparisons test, *N* = 13/14 cells from 3 mice per group; *F* = 0.4560, *p* = 0.7142. NS, not significant. **(F)** One‐way ANOVA with Bonferroni's multiple comparisons test, *N* = 13/14 cells from 3 mice per group; *F* = 1.661, *p* = 0.1873. NS, not significant. **(G‐H)** Cumulative plots of sIPSC interevent intervals **(G)** and amplitude **(H)**. **(I‐J)** Quantification of mean values of sIPSC frequency **(I)** and amplitude **(J)**. **(I)** One‐way ANOVA with Bonferroni's multiple comparisons test, *N* = 13/14 cells from 3 mice per group; *F* = 3.952, *p < 0.05. (J) One‐way ANOVA with Bonferroni's multiple comparisons test, *N* = 13/14 cells from 3 mice per group; *F* = 0.1475, *p* = 0.9308. NS, not significant. **(K)** Quantification of sEPSCs from four groups. One‐way ANOVA with Bonferroni's multiple comparisons test, *N* = 13/14 cells from 3 mice per group; *F* = 0.2354, *p* = 0.8713. NS, not significant. **(L)** Quantification of sIPSCs from four groups. One‐way ANOVA with Bonferroni's multiple comparisons test, *N* = 13/14 cells from 3 mice per group; *F* = 3.630, *p < 0.05. **(M)** Quantification of sEPSC/sIPSC charge transfer ratios. One‐way ANOVA with Bonferroni's multiple comparisons test, *N* = 13/14 cells from 3 mice per group; *F* = 4.567, *p < 0.05, **p < 0.01. Data are mean ± SEM. *N* indicates the number of biologically independent samples, mice per group.

Excitatory and inhibitory (E/I) imbalance plays a critical role in several neuropsychiatric disorders, including schizophrenia. To investigate the impact of EA on synaptic transmission, we performed whole‐cell patch‐clamp recordings from pyramidal neurons in the hippocampal CA1 region (Figure 2A). We measured spontaneous excitatory postsynaptic currents (sEPSCs) and spontaneous inhibitory postsynaptic currents (sIPSCs) to assess potential alterations in synaptic activity. The frequency and amplitude of sEPSCs were similar across all groups, indicating that neither nNos deletion nor EA treatment affected spontaneous glutamatergic transmission (Figure 2B–F). However, we observed that nNos deletion reduced the frequency of sIPSCs in Erbb4‐nNos^−/−^ mice, and this reduction was reversed by EA treatment in the Erbb4‐nNos^−/−^ + EA group (Figure 2B,G,I). No significant differences in sIPSC amplitude were found between the groups (Figure 2B,H,J). To further evaluate the E/I balance, we analyzed the E/I ratio, since both sEPSCs and sIPSCs were recorded from the same neuron. We found no significant difference in sEPSCs among the groups (Figure 2K). While nNos deletion reduced sIPSCs in Erbb4‐nNos^−/−^ mice, EA treatment led to only a trend toward an increase in the Erbb4‐nNos^−/−^ + EA group, without reaching statistical significance (Figure 2L). Notably, the E/I ratio in Erbb4‐nNos^−/−^ mice was higher than that in control mice, and EA treatment in the Erbb4‐nNos^−/−^ + EA group lowered this ratio. These findings suggest that the deletion of nNos from Erbb4‐positive neurons impairs GABAergic transmission and induces an E/I imbalance in the hippocampus, which can be partially restored by EA.

### 3.3 EA Reverses Impaired Basal GABAergic Transmission but Not Basal Excitatory Neurotransmission

**FIGURE 3:**
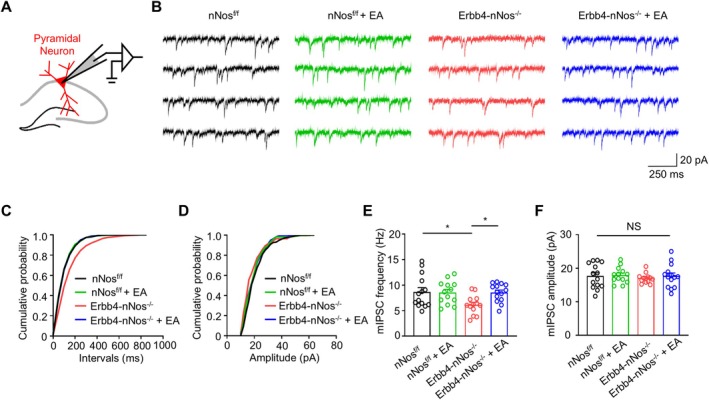
Impaired GABAergic transmission in Erbb4‐nNos^−/−^ mice is reversed by EA. **(A)** Schematic representation of whole‐cell recordings from pyramidal neurons in the hippocampus. **(B)** Representative traces of mIPSCs in CA1 pyramidal neurons from four groups, including nNos^f/f^, nNos^f/f^ + EA, Erbb4‐nNos^−/−^ and Erbb4‐nNos^−/−^ + EA groups. Scale bar = 250 ms, 20 pA. **(C‐D)** Cumulative plots of mIPSC interevent intervals **(C)** and amplitude **(D)**. **(E‐F)** Quantification of mean values of mIPSC frequency **(E)** and amplitude **(F)**. **(E)** One‐way ANOVA with Bonferroni's multiple comparisons test, *N* = 13/14 cells from 3 mice per group; *F* = 3.606, *p* = 0.0195. **(F)** One‐way ANOVA with Bonferroni's multiple comparisons test, *N* = 13/14 cells from 3 mice per group; *F* = 0.2489, *p* = 0.8617. NS, not significant. Data are mean ± SEM. *N* indicates the number of biologically independent samples, mice per group.

To further investigate the impact of EA on basal synaptic transmission in the hippocampus, we first measured miniature excitatory postsynaptic currents (mEPSCs) in pyramidal neurons. We found that both the frequency and amplitude of mEPSCs were comparable across all groups, suggesting that neither nNos deletion nor EA treatment affected basal glutamatergic transmission (Figure S3). Next, we assessed the basal inhibitory synaptic transmission by recording miniature inhibitory postsynaptic currents (mIPSCs). As anticipated, nNos deletion significantly reduced the frequency of mIPSCs, although the amplitude remained unchanged compared to controls. In contrast, EA treatment increased the frequency of mIPSCs (Figure 3A–F). These results suggest that EA may enhance GABA release through a presynaptic mechanism. In summary, our findings indicate that nNos downregulation impairs basal GABAergic transmission but does not affect glutamatergic transmission in hippocampal CA1 neurons. Furthermore, EA treatment can partially restore the impaired GABAergic transmission.

### 3.4 EA Decreases the Cell‐Intrinsic Excitability of Pyramidal Neurons in Erbb4‐nNos ^−/−^ Mice

**FIGURE 4:**
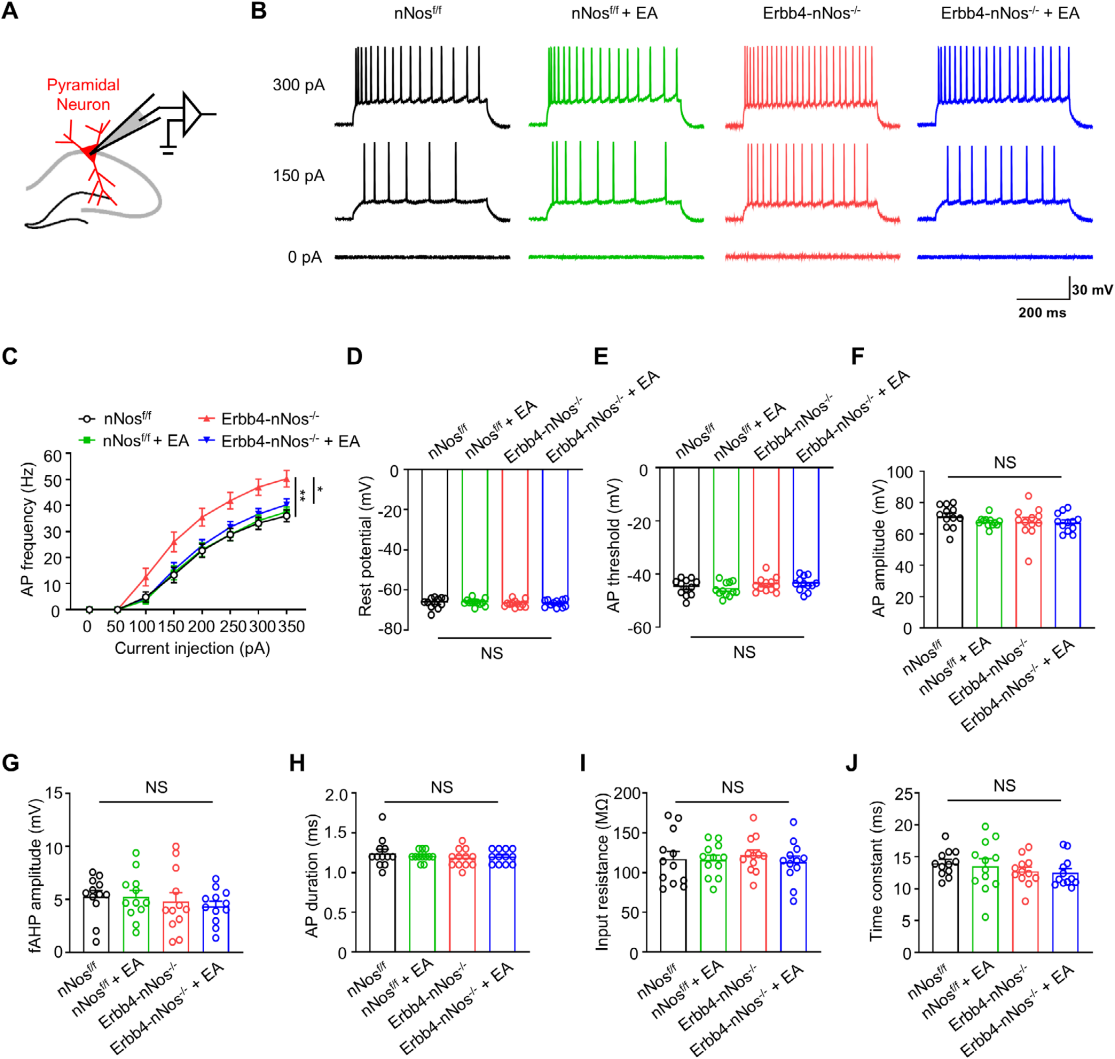
EA decreases the cell‐intrinsic excitability of pyramidal neurons in Erbb4‐nNos^−/−^ mice. **(A)** Schematic of whole‐cell recordings from pyramidal neurons in hippocampus. **(B)** Representative traces of APs in CA1 pyramidal neurons from different groups. Scale bar = 200 ms, 30 mV. **(C)** Quantification of AP frequency induced by different intensity of current injection. Two‐way Repeated Measures ANOVA with Bonferroni's multiple comparisons test, *N* = 12 cells from 3 mice per group; *F* = 5.961, *p < 0.05, **p < 0.01. **(D)** Quantification of rest potential. One‐way ANOVA with Bonferroni's multiple comparisons test, *N* = 12 cells from 3 mice per group; *F* = 0.2706, *p* = 0.8462. NS, not significant. **(E)** Quantification of AP threshold. One‐way ANOVA with Bonferroni's multiple comparisons test, *N* = 12 cells from 3 mice per group; *F* = 1.480, *p* = 0.2330. NS, not significant. **(F)** Quantification of AP amplitude. One‐way ANOVA with Bonferroni's multiple comparisons test, *N* = 12 cells from 3 mice per group; *F* = 0.8485, *p* = 0.4749. NS, not significant. **(G)** Quantification of fAHP amplitude. One‐way ANOVA with Bonferroni's multiple comparisons test, *N* = 12 cells from 3 mice per group; *F* = 0.4317, *p* = 0.7314. NS, not significant. **(H)** Quantification of AP duration. One‐way ANOVA with Bonferroni's multiple comparisons test, *N* = 12 cells from 3 mice per group; *F* = 0.5167, *p* = 0.6729. NS, not significant. **(I)** Quantification of input resistance. One‐way ANOVA with Bonferroni's multiple comparisons test, *N* = 12 cells from 3 mice per group; *F* = 0.2315, *p* = 0.8740. NS, not significant. **(J)** Quantification of time constant. One‐way ANOVA with Bonferroni's multiple comparisons test, *N* = 12 cells from 3 mice per group; *F* = 0.7692, *p* = 0.5174. NS, not significant. Data are mean ± SEM. *N* indicates the number of biologically independent samples, mice per group.

Impaired inhibitory synaptic transmission in the brain has been linked to disinhibition of local excitatory neurons, which can enhance neuronal excitability. To examine how nNos deletion and EA treatment affect the intrinsic excitability of pyramidal neurons, we performed whole‐cell current‐clamp recordings in CA1 pyramidal neurons (Figure 4A,B). Specifically, we assessed action potential (AP) firing rate and conducted a detailed electrophysiological analysis. In Erbb4‐nNos^−/−^ mice, pyramidal neurons exhibited an upward shift in the input–output (I/O) curve for AP firing compared to controls, indicating increased excitability. EA treatment, however, reduced the AP firing rate across various injection currents in the Erbb4‐nNos^−/−^ + EA group (Figure 4C). To investigate the underlying causes of these changes in excitability, we analyzed several electrophysiological parameters, including resting membrane potential (Figure 4D), AP threshold (Figure 4E), AP amplitude (Figure 4F), fast afterhyperpolarization (fAHP) amplitude (Figure 4G), AP duration (Figure 4H), input resistance (Figure 4I), and time constant (τ) (Figure 4J). No significant differences were found across these parameters (Figure 4D–J). In conclusion, nNos downregulation increases the intrinsic excitability of pyramidal neurons, a change that can be attenuated by EA treatment.

### 3.5 EA Enhances Presynaptic GABAergic Transmission in CA1 Pyramidal Neurons From Erbb4‐nNos ^−/−^ Mice

**FIGURE 5:**
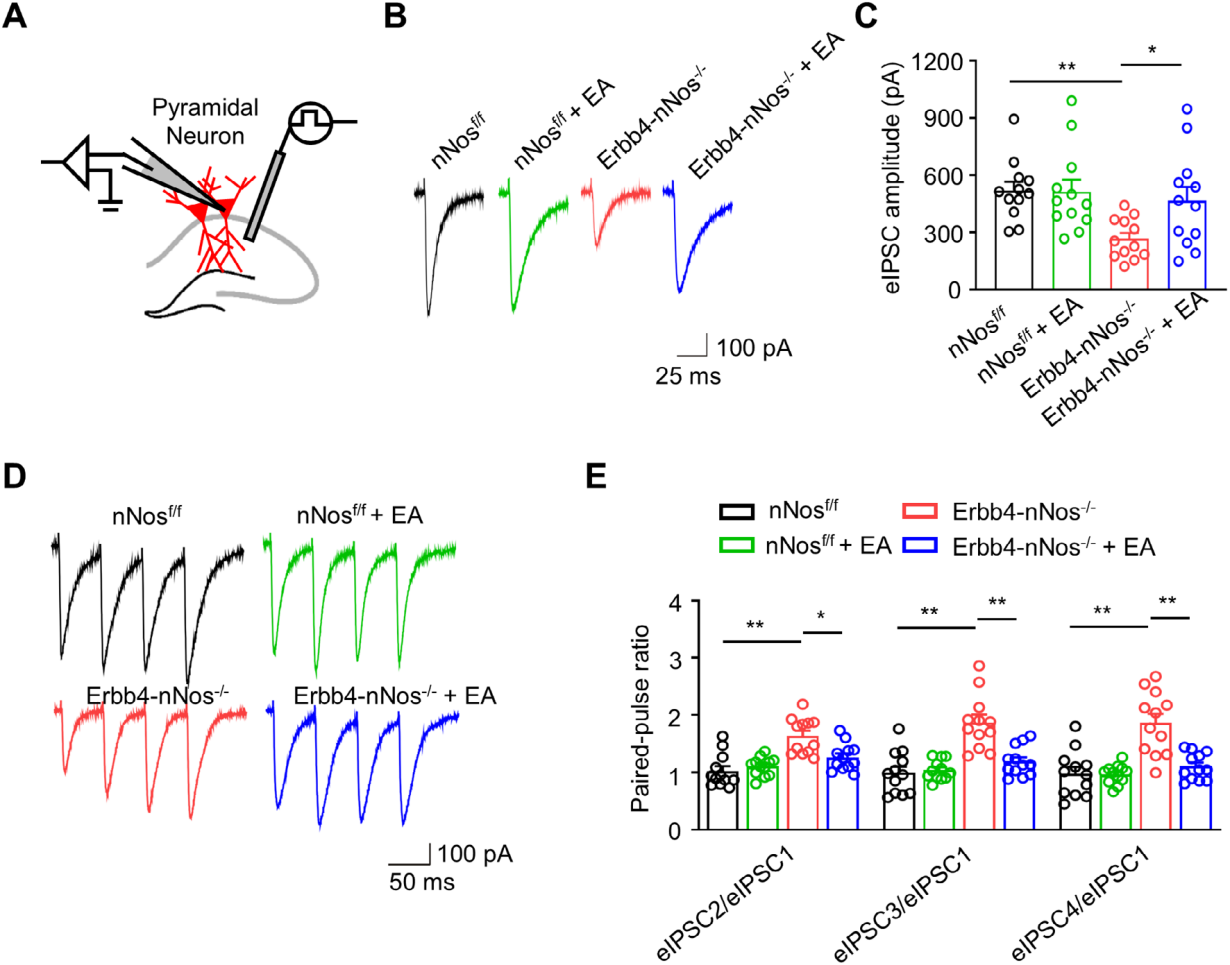
EA enhances presynaptic GABAergic transmission in CA1 pyramidal neurons from Erbb4‐nNos^−/−^ mice. **(A)** Schematic representation of the position of recording from pyramidal neurons and stimulus pipette in hippocampus. **(B‐C)** Representative traces of eIPSCs in CA1 pyramidal neurons from different groups **(B)** and quantification of mean values of eIPSC amplitude **(C)**. Scale bars = 25 ms, 100 pA. **(C)** One‐way ANOVA with Bonferroni's multiple comparisons test, *N* = 12 cells from 3 mice per group; *F* = 4.620, *p < 0.05, **p < 0.01. **(D‐E)** Representative traces **(D)** of PPR of eIPSCs and quantification of mean values of PPR **(E)**. Scale bar = 50 ms, 100 pA. **(E)** Two‐way Repeated Measures ANOVA with Bonferroni's multiple comparisons test, *N* = 12 cells from 3 mice per group; *F* = 18.66, *p < 0.05, **p < 0.01. Data are mean ± SEM. *N* indicates the number of biologically independent samples, mice per group.

In this study, we observed impaired GABAergic transmission, including both spontaneous and basal GABA release. To assess whether nNos deletion and EA treatment affect evoked GABA release, we recorded evoked inhibitory postsynaptic currents (eIPSCs) from CA1 pyramidal neurons in the hippocampus (Figure 5A). We found that the amplitude of eIPSCs was significantly reduced in Erbb4‐nNos^−/−^ mice (Figure 5B,C). Additionally, the deletion of nNos led to an increased paired‐pulse ratio (PPR) of eIPSCs in these mice, suggesting a decrease in release probability (Figure 5D,E). EA treatment reversed this effect by increasing eIPSC amplitude and decreasing the PPR, indicating that EA enhances GABA release through a presynaptic mechanism (Figure 5C,E). Overall, these results demonstrate that nNos deletion impairs GABAergic transmission, a defect that can be partially restored by EA through enhanced presynaptic GABA release.

### 3.6 Inhibition of nNOS Blocks Protective Effects of EA in Erbb4‐nNos ^−/−^ Mice

**FIGURE 6:**
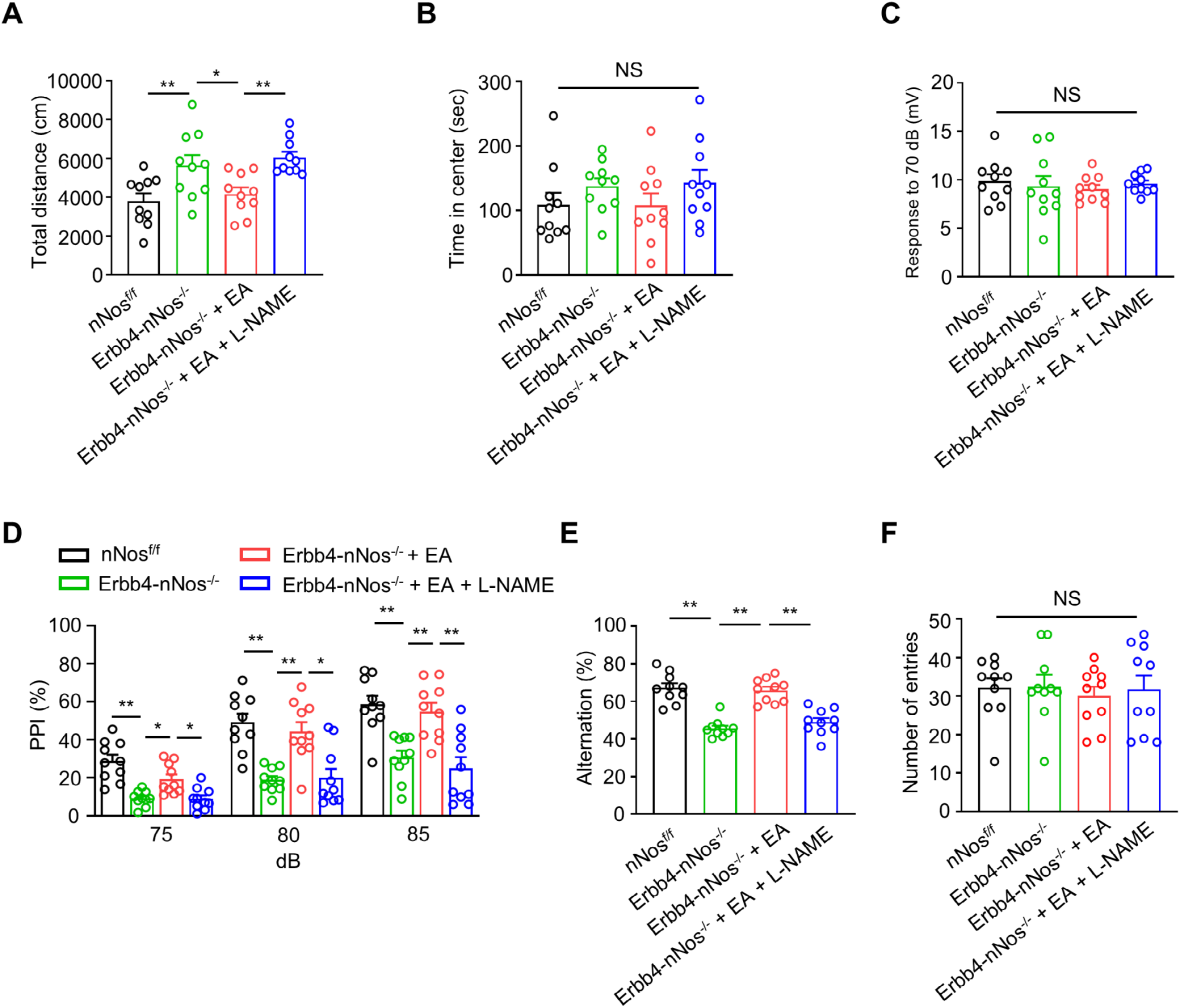
Application of NOS inhibitor, L‐NAME, blocks the protective effect of EA in Erbb4‐nNos^−/−^ mice. **(A‐B)** Total distance **(A)** and time in center **(B)** from the different groups, including nNos^f/f^, Erbb4‐nNos^−/−^, Erbb4‐nNos^−/−^ + EA and Erbb4‐nNos^−/−^ + EA + L‐NAME groups, in open field test. **(A)** One‐way ANOVA with Bonferroni's multiple comparisons test, *N* = 10 per group; *F* = 7.408, *p < 0.05, **p < 0.01. **(B)** One‐way ANOVA with Bonferroni's multiple comparisons test, *N* = 10 per group; *F* = 1.079, *p* = 0.3703. NS, not significant. **(C‐D)** Quantification of response to 70 dB **(C)** and percentage of PPI **(D)** in prepulse inhibition test. **(C)** One‐way ANOVA with Bonferroni's multiple comparisons test, *N* = 10 per group; *F* = 0.2572, *p* = 0.8557. NS, not significant. **(D)** Two‐way Repeated Measures ANOVA with Bonferroni's multiple comparisons test, *N* = 12 per group; *F* = 20.30, *p < 0.05, **p < 0.01. **(E‐F)** Percentage of spontaneous alternation **(E)** and the number of arm entries **(F)** in Y maze. **(E)** One‐way ANOVA with Bonferroni's multiple comparisons test, *N* = 10 per group; *F* = 29.66, **p < 0.01. **(F)** One‐way ANOVA with Bonferroni's multiple comparisons test, *N* = 10 per group; *F* = 0.1405, *p* = 0.9351. NS, not significant. Data are mean ± SEM. *N* indicates the number of biologically independent samples, mice per group.

Previous research has shown that EA can influence nNOS/NO signaling [25], which plays a key role in modulating inhibitory synaptic transmission in the brain [17]. Given that NOS is widely expressed throughout the brain, we aimed to determine whether the NOS/NO system contributes to the therapeutic effects of EA. In the open‐field test, Erbb4‐nNos^−/−^ mice treated with EA exhibited a reduced total distance traveled compared to untreated Erbb4‐nNos^−/−^ mice. However, when treated with L‐NAME, a NOS inhibitor, the total distance increased, suggesting that EA's protective effect on locomotor hyperactivity is dependent on NOS activity (Figure 6A). No significant differences were observed in the time spent in the center area between the groups (Figure 6B). Consistent with previous findings, there were no group differences in the response to background noise (70 dB) (Figure 6C). Notably, the Erbb4‐nNos^−/−^ + EA group showed improved PPI compared to the Erbb4‐nNos^−/−^ group, yet this improvement was completely blocked by L‐NAME (Figure 6D). This indicates that the therapeutic effect of EA on sensorimotor gating is dependent on NOS activity. In the Y‐maze test, the increased percentage of alternation observed in the Erbb4‐nNos^−/−^ + EA group was reduced upon L‐NAME treatment (Figure 6E), although the number of arm entries remained unaffected (Figure 6F). This suggests that NOS inhibition abolishes EA's positive effect on working memory. To determine whether nNOS is sufficient to mediate the EA‐reversed behaviors and synaptic transmission, L‐NPA was administrated to selectively inhibit nNOS activity. Behavioral and electrophysiological results showed that EA failed to ameliorate behavioral deficits and restore the impaired E/I balance in knockout mice (Figures S4 and S5). Taken together, these results demonstrate that nNOS is essential for the therapeutic effects of EA in the Erbb4‐nNos^−/−^ mouse model.

## 4 Discussion

In the mature mammalian brain, the E/I balance is primarily maintained by the neurotransmitters glutamate and GABA, both of which play crucial roles in normal brain function [26]. Studies have indicated that schizophrenia may be characterized by reduced GABAergic transmission and/or increased glutamatergic transmission [27, 28]. Accumulating evidence has demonstrated that hippocampal E/I balance is a common pathological feature of various neuropsychiatric disorders, including schizophrenia [19, 29, 30] and depression [31]. In this study, Erbb4‐nNos^−/−^ mice exhibited behaviors such as hyperactivity, impaired sensorimotor gating, and working memory deficits—phenotypes commonly associated with E/I disruption [19]. This study suggests that the Erbb4‐nNos pathway plays a critical role in modulating E/I balance, and its dysfunction contributes to the schizophrenia‐like traits observed in this model. Overall, this study further supports the pivotal role of E/I imbalance in the pathogenesis of schizophrenia.

Clinical and preclinical studies have consistently supported EA as a promising and safe therapeutic approach for neuropsychiatric disorders, including schizophrenia [32, 33], depression [34, 35] and anxiety [36, 37]. EA therapeutic effects are thought to involve multiple mechanisms, such as reducing neuroinflammation and influencing various signaling pathways [38]. However, the mechanisms underlying the effects of EA on synaptic transmission remain insufficiently explored in the context of schizophrenia. In the present study, EA ameliorated schizophrenia‐like behavioral deficits and restored the excitatory‐inhibitory (E/I) balance, potentially through the enhancement of GABAergic neurotransmission. These findings are of great significance, as hippocampal E/I imbalance induced by reduced GABA release is a well‐established pathological feature of schizophrenia, which is accompanied by excessive pyramidal neuron excitation and consequent cognitive impairments [39, 40]. By boosting GABA levels [41], EA effectively normalizes pyramidal neuron activity [42], thus ameliorating behavioral deficits including cognitive impairments. Furthermore, EA also mitigates the weight gain and lipid metabolism disturbances induced by the antipsychotic drug olanzapine, which may improve treatment adherence in schizophrenia patients [12]. This study provides valuable insights into the neurobiological mechanisms of EA, bridging its clinical applications with underlying synaptic dysfunctions in schizophrenia. These findings emphasize the potential of EA as a therapeutic tool to address core synaptic deficits in neuropsychiatric disorders.

The selection of Baihui (GV20) and Yintang (GV29) as EA acupoints in this study is based on substantial literature and clinical evidence. Specifically, GV20 is a core acupoint for regulating brain function and ameliorating cognitive and emotional symptoms in psychiatric disorders [43]. Both GV20 and GV29 have been used as acupoint combinations in acupuncture interventions for schizophrenia, depression, and anxiety [22, 44, 45], regulating neurotransmitter systems, which is consistent with the E/I balance mechanism investigated herein. Neuroimaging studies demonstrate that acupuncture at GV20 and GV29 specifically enhances functional connectivity between cognitive‐related brain regions, such as the medial prefrontal cortex (mPFC) and the angular gyrus (AG) [46]. Collectively, this evidence confirms that GV20 and GV29 were not randomly selected but represent a specific target combination for schizophrenia‐like cognitive deficits. Meanwhile, EA is less widely applied in the field of schizophrenia than in other psychiatric disorders including anxiety and depression. The intervention protocol (2 Hz, 20 min) used in this study was selected based on the evidence from our previous study in psychiatric disorders [45, 47]. Notably, 1 mA stimulation in mice elicited visible muscle contraction near the acupoints, which is critical for evaluating the therapeutic efficacy. The low‐intensity, safety‐related parameters employed meet clinical EA safety and tolerability requirements. However, differences exist between animals and humans in individual variation, acupoint depth, and stimulation precision. Future large‐scale clinical studies are needed to optimize parameter combinations for different symptom subtypes, facilitating precise translation of EA from basic research to adjunctive clinical therapy for schizophrenia.

In this study, EA significantly ameliorated cognitive and sensorimotor gating deficits such as prepulse inhibition and working memory in schizophrenia model mice, but exerted no significant improvement on social avoidance behavior in the three‐chamber social interaction test. This negative finding suggests that the therapeutic effects of EA are symptom‐selective, rather than broadly effective across all schizophrenia‐like behaviors. Social behavior relies on the coordinated activity of multiple brain regions, including the prefrontal cortex, amygdala, ventral tegmental area, and reward circuits, with neural mechanisms involving multiple systems (e.g., dopamine and oxytocin) [48, 49] that are anatomically and functionally distinct from hippocampus‐dependent cognitive functions. Previous systematic review has shown heterogeneous effects of acupuncture on negative symptoms of schizophrenia [50], including insufficient evidence for the enhancement of social function. Taken together, our findings demonstrate that EA restored E/I balance in the hippocampus but failed to ameliorate social deficits. This discrepancy may be attributed to the more advanced and complex regulatory mechanisms underlying social behavior, as well as the closer association between E/I balance and the positive symptoms of schizophrenia. This evidence suggests that future studies should optimize acupoint combinations or combine EA with other interventions to expand its therapeutic effects on social function.

Electrophysiological results revealed that the frequency of sIPSCs exhibited only an upward trend without statistical significance following EA treatment, whereas the E/I charge ratio was significantly decreased. However, L‐NPA, a nNOS inhibitor, decreased sIPSC frequency and charge transfer of inhibitory synaptic currents in Erbb4‐nNos−/− mice after EA treatment, resulting in an increase in the E/I ratio. In the present study, the E/I ratio may be determined by the frequency of sIPSCs and the integrated total charge transfer of inhibitory synaptic currents within the recording time window. Similar analytical approaches and experimental results have been reported in previous studies of various other diseases, including schizophrenia and anxiety disorders [19, 51]. Individual synaptic parameters (e.g., frequency) are susceptible to random noise and sample heterogeneity, whereas the integrated E/I ratio more stably reflects the overall excitatory‐inhibitory balance of neurons. Previous electrophysiological studies have also confirmed that significant changes in the E/I ratio can arise from the superposition of non‐significant trends in individual synaptic indices, and such integrative analysis better reveals the true functional alterations in synaptic transmission [52, 53]. Moreover, this study demonstrated differences in the firing frequency of current‐evoked action potentials, whereas no significant differences were observed in neuronal membrane properties. Such a difference might be attributed to the fact that altered local inhibitory synaptic transmission regulates the excitability of pyramidal neurons [54]. Above results suggest that EA regulates the E/I balance of hippocampal neurons through synergistic modulation of different inhibitory synaptic parameters (i.e., frequency and charge), thereby providing an electrophysiological basis for its amelioration of certain schizophrenia‐like behavioral deficits.

NOS are classified into three main types: neuronal NOS (nNOS), inducible NOS (iNOS), and endothelial NOS (eNOS). It is worth noting that EA exerts a bidirectional regulatory influence on the activity of NOS and NO production under varying disease conditions [55]. For instance, EA reduces iNOS expression in acute postoperative pain [56] and chemotherapy‐induced peripheral neuropathy [57]. In contrast, EA enhances gastrointestinal function by increasing nNOS expression [58]. In our study, the inhibition of EA's therapeutic effects by L‐NAME, a NOS inhibitor, highlights the crucial role of NOS signaling in the central nervous system for EA's therapeutic efficacy. NO, produced by nNOS, is known to regulate neurotransmitter release, synaptic plasticity, neuronal excitability and various cognitive functions including recognition, learning and memory [17, 59]. Emerging research has linked the nNOS/NO pathway to several neuropsychiatric disorders [60, 61]. Disruption of nNOS‐mediated interactions has been implicated in conditions like depression, anxiety, and addiction [62, 63]. In our study, deletion of nNOS in Erbb4^+^ GABAergic neurons in mice led to schizophrenia‐like behaviors. Furthermore, EA efficacy was abolished following nNOS deletion in Erbb4^+^ interneurons, and residual nNOS inhibition by L‐NAME and L‐NPA abolished the therapeutic effects of EA on these behavioral deficits, suggesting that nNOS from at least two sources contributes to the observed effects. It is well‐established that nNOS is expressed in certain GABAergic interneurons within the hippocampus and neocortex [64]. One possibility is that nNOS, expressed in Erbb4‐negative inhibitory neurons [65] or pyramidal neurons [66] mediates the therapeutic effects by EA. Previous studies demonstrate that NO enhances GABAergic transmission in the inner retina [67] and the brain [68]. Recent work also shows that NO influences mEPSCs in CA1 pyramidal neurons, with nNOS activity being crucial for maintaining excitatory synaptic transmission [69]. These findings underscore the role of nNOS/NO in neurotransmission, though further studies are needed to clarify how nNOS is regulated by EA in a cell‐specific manner.

This study has several limitations that should be acknowledged. First, only male mice were employed in the experiments, without inclusion of female subjects. The use of a single‐sex animal model may compromise the clinical translational relevance of the present findings. Future studies should incorporate both male and female mice to systematically evaluate sex‐specific differences in the therapeutic effects of EA and nNOS‐mediated signaling pathways, thereby enhancing the generalizability of the results. Second, electrophysiological recordings were restricted to the hippocampal CA1 subregion. Schizophrenia is a multifaceted neuropsychiatric disorder involving dysfunction across multiple brain regions, including the prefrontal cortex. In the present study, EA significantly ameliorated prepulse inhibition and working memory, functions that rely heavily on the integrity of the prefrontal cortex and hippocampus. Accordingly, focusing solely on the hippocampus is insufficient to fully characterize the global mechanisms underlying the effects of EA. Further research should therefore extend the analysis to additional brain regions, with particular emphasis on E/I balance, synaptic function, and nNOS signaling in the prefrontal cortex, to clarify the multi‐circuit mechanisms of EA action. Collectively, the present study provides preliminary evidence that nNOS serves as a key molecular target through which EA restores hippocampal E/I balance. Nevertheless, future investigations are warranted to address sex differences and multi‐regional mechanisms, so as to furnish more comprehensive and rigorous preclinical evidence to support the clinical translation of EA for the treatment of schizophrenia.

## 5 Conclusions

In summary, our results suggest that EA alleviates schizophrenia‐related behavioral deficits in Erbb4‐nNos^−/−^ mice, which can be blocked by the nNOS inhibitor, with its therapeutic effects mediated by restoring the E/I balance through the enhancement of presynaptic GABAergic transmission. This finding underscores the role of the nNOS/NO system in the therapeutic effects of EA, highlighting that maintaining or restoring this pathway is crucial for EA‐mediated neuroprotection. These insights advance our understanding of the neurophysiological mechanisms underlying EA's potential benefits for treating neuropsychiatric disorders.

## Author Contributions

Yongjun Chen and Lin Yao designed the experiments. Yongjun Chen, Lin Yao, and Chaofan Wan wrote the manuscript. Chaofan Wan, Yucen Xia, Jinglan Yan, Weipeng Lin, Yuanjia Zheng, and Yiqiu Lin performed the electrophysiological and behavioral experiments and analyzed the data. All authors read and approved the final manuscript.

## Funding

This work was supported by the National Natural Science Foundation of China (82205272 and 82505776), Key R&D Program of Shandong Province, China (2025CXPT134), the National Comprehensive Traditional Chinese Medicine Reform Demonstration Zone Science and Technology Collaborative Development Project (GZY‐KJS‐SD‐2024‐046). This work was also supported, in part, by the Guangdong Medical Research Foundation (A2024184) and the Youth Team Project of Guangdong Pharmaceutical University (2024QZ14).

## Ethics Statement

The animal experiments were approved by the Animals Care and Use Committee of Guangzhou University of Chinese Medicine (NO. 00226066) and followed the National Institutes of Health Guidelines for the Care and Use of Laboratory Animals.

## Conflicts of Interest

The authors declare no conflicts of interest.

## Supporting information


**Figure S1:** EA has no effect on time in center in open field test and latency to fall in rotarod test. **(A)** Scheme of experimental design. Breeding diagram for the generation of Erbb4^CreER/+^; nNos^f/f^ mice (Erbb4‐nNos^−/−^ mice). **(B)** Quantification of time in center from different groups in open field test. One‐way ANOVA with Bonferroni's multiple comparisons test, *N* = 12 per group; *F* = 0.3625, *p* = 0.7804. NS, not significant. **(C)** Quantification of latency to fall in rotarod test. Repeated two‐way ANOVA with Bonferroni's multiple comparisons test, *N* = 12 per group; *F* = 0.2441, *p* = 0.8651. Data are mean ± SEM. *N* indicates the number of biologically independent samples, mice per group.
**Figure S2:** Acupuncture improves schizophrenia‐relevant behavioral deficits in Erbb4‐nNos^−/−^ mice. **(A‐B)** Total distance **(A)** and time in center **(B)** from the different groups, including Erbb4‐nNos^−/−^, Erbb4‐nNos^−/−^ + Sham and Erbb4‐nNos^−/−^ + AC groups, in open field test. **(A)** One‐way ANOVA with Bonferroni's multiple comparisons test, *N* = 7–8 per group; *F* = 5.520, *p < 0.05. **(B)** One‐way ANOVA with Bonferroni's multiple comparisons test, *N* = 7–8 per group; *F* = 2.797, *p* = 0.0862. NS, not significant. **(C‐D)** Quantification of response to 70 dB **(C)** and percentage of PPI **(D)** in prepulse inhibition test. **(C)** One‐way ANOVA with Bonferroni's multiple comparisons test, *N* = 7–8 per group; *F* = 0.8013, *p* = 0.4633. NS, not significant. **(D)** Two‐way Repeated Measures ANOVA with Bonferroni's multiple comparisons test, *N* = 7–8 per group; *F* = 32.16, **p < 0.01. **(E‐F)** Percentage of spontaneous alternation **(E)** and the number of arm entries **(F)** in Y maze. **(E)** One‐way ANOVA with Bonferroni's multiple comparisons test, *N* = 7–8 per group; *F* = 5.291, *p < 0.01. **(F)** One‐way ANOVA with Bonferroni's multiple comparisons test, *N* = 7–8 per group; *F* = 0.0572, *p* = 0.9445. NS, not significant. Data are mean ± SEM. *N* indicates the number of biologically independent samples, mice per group.
**Figure S3:** EA has little effect on basal excitatory neurotransmission in the hippocampus from Erbb4‐nNos^−/−^ mice. **(A)** Schematic representation of whole‐cell recordings from pyramidal neurons in hippocampus. **(B)** Representative traces of mEPSCs in CA1 pyramidal neurons from four groups, including nNos^f/f^, nNos^f/f^ + EA, Erbb4‐nNos^−/−^ and Erbb4‐nNos^−/−^ + EA groups. Scale bar = 250 ms, 15 pA. **(C‐D)** Cumulative plots of mEPSC interevent intervals **(C)** and amplitude **(D)**. **(E‐F)** Quantification of mean values of mEPSC frequency **(E)** and amplitude **(F)**. **(E)** One‐way ANOVA with Bonferroni's multiple comparisons test, *N* = 13/14 cells from 3 mice per group; *F* = 0.02459, *p* = 0.9947. NS, not significant. **(F)** One‐way ANOVA with Bonferroni's multiple comparisons test, *N* = 13/14 cells from 3 mice per group; *F* = 0.4123, *p* = 0.7449. NS, not significant. Data are mean ± SEM. *N* indicates the number of biologically independent samples, mice per group.
**Figure S4:** L‐NPA, a selective nNOS inhibitor, abolishes the therapeutic effect of EA in Erbb4‐nNos^−/−^ mice. **(A‐B)** Total distance **(A)** and time in center **(B)** from two groups, including Erbb4‐nNos^−/−^ + EA and Erbb4‐nNos^−/−^ + EA + L‐NPA groups, in open field test. **(A)** Unpaired *t* test, *N* = 8–9 per group; *p* = 0.0299, *p < 0.05. **(B)** Unpaired *t* test, *N* = 8–9 per group; *p* = 0.1785. NS, not significant. **(C‐D)** Quantification of response to 70 dB **(C)** and percentage of PPI **(D)** in prepulse inhibition test. **(C)** Unpaired *t* test, *N* = 8–9 per group; *p* = 0.4153. NS, not significant. **(D)** Two‐way Repeated Measures ANOVA with Bonferroni's multiple comparisons test, *N* = 8–9 per group; *F* = 16.97, *p < 0.05, **p < 0.01. **(E‐F)** Percentage of spontaneous alternation **(E)** and the number of arm entries **(F)** in Y maze. **(E)** Unpaired t test, *N* = 8–9 per group; *p* = 0.0256, *p < 0.01. **(F)** Unpaired *t* test, *N* = 8–9 per group; *F* = 0.1405, *p* = 0.9807. NS, not significant. Data are mean ± SEM. *N* indicates the number of biologically independent samples, mice per group.
**Figure S5:** L‐NPA blocks the therapeutic effect of EA on E/I balance in Erbb4‐nNos^−/−^ mice. **(A)** Schematic representation of whole‐cell recordings from pyramidal neurons in hippocampus. **(B)** Representative traces of sEPSCs (−60 mV) and sIPSCs (+10 mV) in CA1 pyramidal neurons in two groups, including Erbb4‐nNos^−/−^ + EA and Erbb4‐nNos^−/−^ + EA + L‐NPA groups. Scale bar = 2 s, 10 pA. **(C‐D)** Quantification of mean values of sEPSC frequency **(C)** and amplitude **(D)**. **(C)** Unpaired t test, *N* = 10 cells from 3 mice per group; *p* = 0.7779. NS, not significant. **(D)** Unpaired *t* test, *N* = 10 cells from 3 mice per group; *p* = 0.0850. NS, not significant. **(E‐F)** Quantification of mean values of sIPSC frequency **(E)** and amplitude **(F)**. **(E)** Unpaired t test, *N* = 10 cells from 3 mice per group; *p* = 0.0098. **p < 0.01. **(F)** Unpaired *t* test, *N* = 10 cells from 3 mice per group; *p* = 0.1241. NS, not significant. **(G)** Quantification of sEPSCs from four groups. Unpaired *t* test, *N* = 10 cells from 3 mice per group; *p* = 0.6442. NS, not significant. **(H)** Quantification of sIPSCs from four groups. Unpaired t test, *N* = 10 cells from 3 mice per group; *p* = 0.0046. **p < 0.01. **(I)** Quantification of sEPSC/sIPSC charge transfer ratios. Unpaired t test, *N* = 10 cells from 3 mice per group; *p* = 0.0417. *p < 0.05. Data are mean ± SEM. *N* indicates the number of biologically independent samples, mice per group.

## Data Availability

The data that support the findings of this study are available from the corresponding author upon reasonable request.

## References

[1] O. D. Howes , B. R. Bukala , and K. Beck , “Schizophrenia: From Neurochemistry to Circuits, Symptoms and Treatments,” Nature Reviews. Neurology 20, no. 1 (2024): 22–35. 38110704 10.1038/s41582-023-00904-0

[2] S. Jauhar , M. Johnstone , and P. J. McKenna , “Schizophrenia,” Lancet 399, no. 10323 (2022): 473–486. 35093231 10.1016/S0140-6736(21)01730-X

[3] M. Solmi , G. Seitidis , D. Mavridis , et al., “Incidence, Prevalence, and Global Burden of Schizophrenia ‐ Data, With Critical Appraisal, From the Global Burden of Disease (GBD) 2019,” Molecular Psychiatry 28, no. 12 (2023): 5319–5327. 37500825 10.1038/s41380-023-02138-4

[4] A. de Bartolomeis , G. De Simone , M. De Prisco , et al., “Insulin Effects on Core Neurotransmitter Pathways Involved in Schizophrenia Neurobiology: A Meta‐Analysis of Preclinical Studies. *Implications for the Treatment* ,” Molecular Psychiatry 28, no. 7 (2023): 2811–2825. 37085712 10.1038/s41380-023-02065-4 PMC10615753

[5] O. D. Howes , E. Dawkins , M. C. Lobo , S. J. Kaar , and K. Beck , “New Drug Treatments for Schizophrenia: A Review of Approaches to Target Circuit Dysfunction,” Biological Psychiatry 96, no. 8 (2024): 638–650. 38815885 10.1016/j.biopsych.2024.05.014

[6] S. Dong , L. Zhao , J. Liu , et al., “Neuroanatomical Organization of Electroacupuncture in Modulating Gastric Function in Mice and Humans,” Neuron 113 (2025): 3243–3259.e11. 40713955 10.1016/j.neuron.2025.06.023

[7] S. Liu , Z. Wang , Y. Su , et al., “A Neuroanatomical Basis for Electroacupuncture to Drive the Vagal‐Adrenal Axis,” Nature 598, no. 7882 (2021): 641–645. 34646018 10.1038/s41586-021-04001-4 PMC9178665

[8] X. Zheng , W. Lin , Y. Jiang , et al., “Electroacupuncture Ameliorates Beta‐Amyloid Pathology and Cognitive Impairment in Alzheimer Disease via a Novel Mechanism Involving Activation of TFEB (Transcription Factor EB),” Autophagy 17, no. 11 (2021): 3833–3847. 33622188 10.1080/15548627.2021.1886720 PMC8632298

[9] Y. Xin , S. Zhou , T. Chu , Y. Zhou , and A. Xu , “Protective Role of Electroacupuncture Against Cognitive Impairment in Neurological Diseases,” Current Neuropharmacology 23, no. 2 (2025): 145–171. 38379403 10.2174/1570159X22999240209102116 PMC11793074

[10] T. Tong , C. Hao , J. Shen , et al., “Electroacupuncture Ameliorates Chronic Unpredictable Mild Stress‐Induced Depression‐Like Behavior and Cognitive Impairment Through Suppressing Oxidative Stress and Neuroinflammation in Rats,” Brain Research Bulletin 206 (2024): 110838. 38123022 10.1016/j.brainresbull.2023.110838

[11] Z. Zhang , W. Lin , J. Yan , et al., “Prenatal Electroacupuncture Modulates Maternal‐Fetal Immune Activation via a Brain‐To‐Splenic Signal,” Cell Reports 44, no. 11 (2025): 116576. 41259207 10.1016/j.celrep.2025.116576

[12] J. Chen , P. Ju , C. Luo , et al., “Effect of Electroacupuncture at Zusanli Reduced Olanzapine‐Induced Lipid Disturbances in Mice via Potential Liver‐Brain Interaction,” FASEB Journal 39, no. 8 (2025): e70491. 40235271 10.1096/fj.202402319R

[13] M. J. Owen , S. E. Legge , E. Rees , J. T. R. Walters , and M. C. O'Donovan , “Genomic Findings in Schizophrenia and Their Implications,” Molecular Psychiatry 28, no. 9 (2023): 3638–3647. 37853064 10.1038/s41380-023-02293-8 PMC10730422

[14] J. Huang , Y. Y. Zhang , Y. Y. Qiu , et al., “NRG1‐ErbB4 Signaling in the Medial Amygdala Controls Mating Motivation in Adult Male Mice,” Cell Reports 43, no. 3 (2024): 113905. 38446660 10.1016/j.celrep.2024.113905

[15] H. Wang , F. Liu , W. Chen , et al., “Genetic Recovery of ErbB4 in Adulthood Partially Restores Brain Functions in Null Mice,” Proceedings of the National Academy of Sciences of the United States of America 115, no. 51 (2018): 13105–13110. 30498032 10.1073/pnas.1811287115 PMC6304932

[16] A. Kiemes , M. E. Serrano Navacerrada , E. Kim , et al., “Erbb4 Deletion From Inhibitory Interneurons Causes Psychosis‐Relevant Neuroimaging Phenotypes,” Schizophrenia Bulletin 49, no. 3 (2023): 569–580. 36573631 10.1093/schbul/sbac192 PMC10154722

[17] L. J. Zhu , F. Li , and D. Y. Zhu , “nNOS and Neurological, Neuropsychiatric Disorders: A 20‐Year Story,” Neuroscience Bulletin 39, no. 9 (2023): 1439–1453. 37074530 10.1007/s12264-023-01060-7 PMC10113738

[18] H. Baba , T. Suzuki , H. Arai , and P. C. Emson , “Expression of nNOS and Soluble Guanylate Cyclase in Schizophrenic Brain,” Neuroreport 15, no. 4 (2004): 677–680. 15094474 10.1097/00001756-200403220-00020

[19] C. Wan , Y. Xia , J. Yan , et al., “nNOS in Erbb4‐Positive Neurons Regulates GABAergic Transmission in Mouse Hippocampus,” Cell Death & Disease 15, no. 2 (2024): 167. 38396027 10.1038/s41419-024-06557-1 PMC10891175

[20] K. A. Hyndman , E. I. Boesen , A. A. Elmarakby , et al., “Renal Collecting Duct NOS1 Maintains Fluid‐Electrolyte Homeostasis and Blood Pressure,” Hypertension 62, no. 1 (2013): 91–98. 23608660 10.1161/HYPERTENSIONAHA.113.01291 PMC3901402

[21] C. Wan , Y. Xu , B. Cen , et al., “Neuregulin1‐ErbB4 Signaling in Spinal Cord Participates in Electroacupuncture Analgesia in Inflammatory Pain,” Frontiers in Neuroscience 15 (2021): 636348. 33584196 10.3389/fnins.2021.636348 PMC7875897

[22] L. Chen , Z. Liu , Z. Zhao , et al., “Dopamine Receptor 1 on CaMKII‐Positive Neurons Within Claustrum Mediates Adolescent Cocaine Exposure‐Induced Anxiety‐Like Behaviors and Electro‐Acupuncture Therapy,” Theranostics 13, no. 10 (2023): 3149–3164. 37351159 10.7150/thno.83079 PMC10283049

[23] L. Yao , Q. Ye , Y. Liu , et al., “Electroacupuncture Improves Swallowing Function in a Post‐Stroke Dysphagia Mouse Model by Activating the Motor Cortex Inputs to the Nucleus Tractus Solitarii Through the Parabrachial Nuclei,” Nature Communications 14, no. 1 (2023): 810. 10.1038/s41467-023-36448-6 PMC9925820 36781899

[24] J. Tong , K. Yang , W. Li , et al., “N‐Methyl‐d‐Aspartate Receptor Antibody and Sensory Gating Deficits in Non‐Smoking, Minimal Antipsychotic Medication Exposure, and First‐Episode Patients With Schizophrenia,” Schizophrenia Bulletin 51, no. 4 (2025): 1072–1081. 39406395 10.1093/schbul/sbae180 PMC12236339

[25] C. Zhang , T. Chen , M. Fan , et al., “Electroacupuncture Improves Gastrointestinal Motility Through a Central‐Cholinergic Pathway‐Mediated GDNF Releasing From Intestinal Glial Cells to Protect Intestinal Neurons in Parkinson's Disease Rats,” Neurotherapeutics 21, no. 4 (2024): e00369. 38744625 10.1016/j.neurot.2024.e00369 PMC11305299

[26] Z. P. Chen , X. Zhao , S. Wang , et al., “GABA‐Dependent Microglial Elimination of Inhibitory Synapses Underlies Neuronal Hyperexcitability in Epilepsy,” Nature Neuroscience 28, no. 7 (2025): 1404–1417. 40425792 10.1038/s41593-025-01979-2

[27] L. A. Chamberlin , S. S. Yang , E. P. McEachern , et al., “Pharmacogenetic Activation of Parvalbumin Interneurons in the Prefrontal Cortex Rescues Cognitive Deficits Induced by Adolescent MK801 Administration,” Neuropsychopharmacology 48, no. 9 (2023): 1267–1276. 37041206 10.1038/s41386-023-01576-6 PMC10353985

[28] Y. Xia , Z. Zhang , W. Lin , et al., “Modulating Microglia Activation Prevents Maternal Immune Activation Induced Schizophrenia‐Relevant Behavior Phenotypes via Arginase 1 in the Dentate Gyrus,” Neuropsychopharmacology 45, no. 11 (2020): 1896–1908. 32599605 10.1038/s41386-020-0743-7 PMC7608378

[29] S. Liu , Y. Zhang , K. Liu , et al., “Robo2‐Nrxn3 Deficiency: A Molecular Hub Linking Excitation‐Inhibition Imbalance to the Pathogenesis of Schizophrenia,” Schizophrenia Bulletin 52, no. 2 (2026): sbag005. 41863359 10.1093/schbul/sbag005 PMC13005109

[30] Y. Curto , H. Carceller , P. Klimczak , et al., “Erythropoietin Restrains the Inhibitory Potential of Interneurons in the Mouse Hippocampus,” Molecular Psychiatry 29, no. 10 (2024): 2979–2996. 38622200 10.1038/s41380-024-02528-2 PMC11449791

[31] H. J. Shi , Y. R. Xue , H. Shao , et al., “Hippocampal Excitation‐Inhibition Balance Underlies the 5‐HT2C Receptor in Modulating Depressive Behaviours,” Brain 147, no. 11 (2024): 3764–3779. 38701344 10.1093/brain/awae143

[32] Q. Li , Y. Gong , Y. Cui , et al., “Efficacy of Transcutaneous Electrical Acupoint Stimulation for Patients With First‐Episode Schizophrenia: An 8‐Week, Preliminary, Randomized Controlled Trial,” Psychiatry Research 325 (2023): 115255. 37245485 10.1016/j.psychres.2023.115255

[33] H. Zhaohan , F. Yuan , W. Xiaolu , H. Yue , Y. U. Qi , and W. Tong , “Effectiveness of Acupuncture‐Related Therapies on Schizophrenia: A Bayesian Network Meta‐Analysis,” Journal of Traditional Chinese Medicine 43, no. 2 (2023): 239–251. 36994512 10.19852/j.cnki.jtcm.20221226.001 PMC10012197

[34] X. Yin , W. Li , T. Liang , et al., “Effect of Electroacupuncture on Insomnia in Patients With Depression: A Randomized Clinical Trial,” JAMA Network Open 5, no. 7 (2022): e2220563. 35797047 10.1001/jamanetworkopen.2022.20563 PMC9264041

[35] S. S. Lin , B. Zhou , B. J. Chen , et al., “Electroacupuncture Prevents Astrocyte Atrophy to Alleviate Depression,” Cell Death & Disease 14, no. 5 (2023): 343. 37248211 10.1038/s41419-023-05839-4 PMC10227075

[36] Z. Q. Shen , W. Q. Chang , L. F. Liang , et al., “Electroacupuncture Effects on Trigeminal Neuralgia With Comorbid Anxiety and Depression: The Role of Frequency and Acupoint Specificity,” FASEB Journal 39, no. 2 (2025): e70337. 39840659 10.1096/fj.202402461RR

[37] Y. Chen , S. Tong , Y. Xu , et al., “Involvement of Basolateral Amygdala‐Rostral Anterior Cingulate Cortex in Mechanical Allodynia and Anxiety‐Like Behaviors and Potential Mechanisms of Electroacupuncture,” CNS Neuroscience & Therapeutics 30, no. 9 (2024): e70035. 39279046 10.1111/cns.70035 PMC11402788

[38] X. Han , Y. Gao , X. Yin , et al., “The Mechanism of Electroacupuncture for Depression on Basic Research: A Systematic Review,” Chinese Medicine 16, no. 1 (2021): 10. 33436036 10.1186/s13020-020-00421-y PMC7805231

[39] J. Maksymetz , N. E. Byun , D. J. Luessen , et al., “mGlu(1) Potentiation Enhances Prelimbic Somatostatin Interneuron Activity to Rescue Schizophrenia‐Like Physiological and Cognitive Deficits,” Cell Reports 37, no. 5 (2021): 109950. 34731619 10.1016/j.celrep.2021.109950 PMC8628371

[40] D. Y. Gawande , K. K. S. Narasimhan , G. P. Shelkar , R. Pavuluri , H. A. F. Stessman , and S. M. Dravid , “GluN2D Subunit in Parvalbumin Interneurons Regulates Prefrontal Cortex Feedforward Inhibitory Circuit and Molecular Networks Relevant to Schizophrenia,” Biological Psychiatry 94, no. 4 (2023): 297–309. 37004850 10.1016/j.biopsych.2023.03.020 PMC10524289

[41] J. Li , X. Wu , S. Yan , et al., “Understanding the Antidepressant Mechanisms of Acupuncture: Targeting Hippocampal Neuroinflammation, Oxidative Stress, Neuroplasticity, and Apoptosis in CUMS Rats,” Molecular Neurobiology 62, no. 4 (2025): 4221–4236. 39422855 10.1007/s12035-024-04550-5 PMC11880061

[42] J. Wu , L. Hua , W. Liu , et al., “Electroacupuncture Exerts Analgesic Effects by Restoring Hyperactivity via Cannabinoid Type 1 Receptors in the Anterior Cingulate Cortex in Chronic Inflammatory Pain,” Molecular Neurobiology 61, no. 5 (2024): 2949–2963. 37957422 10.1007/s12035-023-03760-7 PMC11043129

[43] E. Y. Shen , F. J. Chen , Y. Y. Chen , and M. F. Lin , “Locating the Acupoint Baihui (GV20) Beneath the Cerebral Cortex With MRI Reconstructed 3D Neuroimages,” Evidence‐Based Complementary and Alternative Medicine 2011 (2011): 362494. 21785620 10.1093/ecam/neq047 PMC3135375

[44] Z. L. Sun , J. Liu , W. Guo , et al., “Serum Brain‐Derived Neurotrophic Factor Levels Associate With Cognitive Improvement in Patients With Schizophrenia Treated With Electroacupuncture,” Psychiatry Research 244 (2016): 370–375. 27525826 10.1016/j.psychres.2016.07.040

[45] T. Yin , Y. Wang , C.'. Zhu , et al., “Electroacupuncture Attenuates Anxiety Caused by Chronic Mild Stress Through Inhibiting NOX2‐Derived Oxidative Stress in Ventral Hippocampus,” Neurobiology of Stress 39 (2025): 100768. 41280328 10.1016/j.ynstr.2025.100768 PMC12629925

[46] X. Wang , T. Huang , H. Lei , Q. Cui , W. Wei , and H. Lin , “Immediate and Sustained Effects of Acupuncture on the Default Mode Network,” Brazilian Journal of Psychiatry 47 (2025): e20254202. 41022570 10.47626/1516-4446-2025-4202 PMC12815408

[47] L. Guo , X. Liang , Z. Liang , et al., “Electroacupuncture Ameliorates Cognitive Deficit and Improves Hippocampal Synaptic Plasticity in Adult Rat With Neonatal Maternal Separation,” Evidence‐Based Complementary and Alternative Medicine 2018 (2018): 2468105. 29785188 10.1155/2018/2468105 PMC5896274

[48] W. Zhao , X. Zhang , and K. M. Kendrick , “From Fundamental Research to Clinical Translation: The Neural Modulation of Social Behavior by Oxytocin and Vasopressin,” Neuroscience and Biobehavioral Reviews 185 (2026): 106624. 41796878 10.1016/j.neubiorev.2026.106624

[49] S. Fujima and M. Sato , “Oxytocin Modulation of the Insular Cortex: Implications for Social Cognition and Neurodevelopmental Disorders,” Frontiers in Neural Circuits 20 (2026): 1791625. 41970203 10.3389/fncir.2026.1791625 PMC13066175

[50] M. van den Noort , S. Yeo , S. Lim , S. H. Lee , H. Staudte , and P. Bosch , “Acupuncture as Add‐On Treatment of the Positive, Negative, and Cognitive Symptoms of Patients With Schizophrenia: A Systematic Review,” Medicines (Basel) 5, no. 2 (2018): 29. 29601477 10.3390/medicines5020029 PMC6023351

[51] G. Yang , Y. Zhao , C. Zhao , et al., “Kv1.1 Channel Dysfunction in Parvalbumin‐Positive Interneurons Contributes to Anxiety‐Like Behaviors in Young Adult Presenilin 1/2 Conditional Double Knockout Mice,” Cell & Bioscience 15, no. 1 (2025): 89. 40563122 10.1186/s13578-025-01422-w PMC12199491

[52] H. Lv , C. Zhu , R. Wu , et al., “Chronic Mild Stress Induced Anxiety‐Like Behaviors Can be Attenuated by Inhibition of NOX2‐Derived Oxidative Stress,” Journal of Psychiatric Research 114 (2019): 55–66. 31039481 10.1016/j.jpsychires.2019.04.008

[53] H. Li , J. Zhao , L. Lai , et al., “Loss of SST and PV Positive Interneurons in the Ventral Hippocampus Results in Anxiety‐Like Behavior in 5xFAD Mice,” Neurobiology of Aging 117 (2022): 165–178. 35764035 10.1016/j.neurobiolaging.2022.05.013

[54] Y. Y. Wang , B. Zhao , M. M. Wu , X. L. Zheng , L. Lin , and D. M. Yin , “Overexpression of Neuregulin 1 in GABAergic Interneurons Results in Reversible Cortical Disinhibition,” Nature Communications 12, no. 1 (2021): 278. 10.1038/s41467-020-20552-y PMC7804852 33436636

[55] S. X. Ma , “Stimuli‐Evoked NOergic Molecules and Neuropeptides at Acupuncture Points and the Gracile Nucleus Contribute to Signal Transduction of Propagated Sensation Along the Meridian Through the Dorsal Medulla‐Thalamic Pathways,” Journal of Integrative Medicine 22, no. 5 (2024): 515–522. 39214715 10.1016/j.joim.2024.07.001 PMC11439578

[56] Y. Y. Ding , F. Xu , Y. F. Wang , et al., “Electroacupuncture Alleviates Postoperative Pain Through Inhibiting Neuroinflammation via Stimulator of Interferon Genes/Type‐1 Interferon Pathway,” Journal of Integrative Medicine 21, no. 5 (2023): 496–508. 37517892 10.1016/j.joim.2023.07.001

[57] X. Ma , Y. Chen , X. C. Li , et al., “Spinal Neuronal GRK2 Contributes to Preventive Effect by Electroacupuncture on Cisplatin‐Induced Peripheral Neuropathy in Mice,” Anesthesia and Analgesia 134, no. 1 (2022): 204–215. 34652301 10.1213/ANE.0000000000005768 PMC8647702

[58] Y. Zhang , Y. W. Tang , J. Zhou , et al., “Electroacupuncture at ST36 Ameliorates Gastric Dysmotility in Rats With Diabetic Gastroparesis via the Nucleus Tractus Solitarius‐Vagal Axis,” World Journal of Gastroenterology 31, no. 21 (2025): 107395. 40538511 10.3748/wjg.v31.i21.107395 PMC12175861

[59] E. Candemir , N. Fattakhov , A. O. Leary , et al., “Disrupting the nNOS/NOS1AP Interaction in the Medial Prefrontal Cortex Impairs Social Recognition and Spatial Working Memory in Mice,” European Neuropsychopharmacology 67 (2023): 66–79. 36513018 10.1016/j.euroneuro.2022.11.006

[60] H. J. Shi , D. L. Wu , R. Chen , N. Li , and L. J. Zhu , “Requirement of Hippocampal DG nNOS‐CAPON Dissociation for the Anxiolytic and Antidepressant Effects of Fluoxetine,” Theranostics 12, no. 8 (2022): 3656–3675. 35664081 10.7150/thno.70370 PMC9131266

[61] X. H. Zhu , Y. P. Zhou , Q. Zhang , et al., “A Novel Anti‐Epileptogenesis Strategy of Temporal Lobe Epilepsy Based on Nitric Oxide Donor,” EMBO Molecular Medicine 17, no. 1 (2025): 85–111. 39653809 10.1038/s44321-024-00168-1 PMC11730642

[62] N. Sun , Y. J. Qin , C. Xu , et al., “Design of Fast‐Onset Antidepressant by Dissociating SERT From nNOS in the DRN,” Science 378, no. 6618 (2022): 390–398. 36302033 10.1126/science.abo3566

[63] X. Kou , J. Xian , Z. Huang , et al., “Disrupting the Interaction of nNOS With CAPON Prevents the Reinstatement of Morphine Conditioned Place Preference,” Cerebral Cortex 32, no. 3 (2022): 569–582. 34297798 10.1093/cercor/bhab234

[64] L. Tricoire and T. Vitalis , “Neuronal Nitric Oxide Synthase Expressing Neurons: A Journey From Birth to Neuronal Circuits,” Frontiers in Neural Circuits 6 (2012): 82. 23227003 10.3389/fncir.2012.00082 PMC3514612

[65] Z. Christenson Wick , M. R. Tetzlaff , and E. Krook‐Magnuson , “Novel Long‐Range Inhibitory nNOS‐Expressing Hippocampal Cells,” eLife 8 (2019): 8. 10.7554/eLife.46816 PMC6839902 31609204

[66] A. Saunders , E. Z. Macosko , A. Wysoker , et al., “Molecular Diversity and Specializations Among the Cells of the Adult Mouse Brain,” Cell 174, no. 4 (2018): 1015–1030.e16. 30096299 10.1016/j.cell.2018.07.028 PMC6447408

[67] J. W. Maddox , N. Khorsandi , and E. Gleason , “TRPC5 Is Required for the NO‐Dependent Increase in Dendritic ca(2+) and GABA Release From Chick Retinal Amacrine Cells,” Journal of Neurophysiology 119, no. 1 (2018): 262–273. 28978766 10.1152/jn.00500.2017

[68] S. Wang , A. G. Teschemacher , J. F. R. Paton , et al., “Mechanism of Nitric Oxide Action on Inhibitory GABAergic Signaling Within the Nucleus Tractus Solitarii,” FASEB Journal 20, no. 9 (2006): 1537–1539. 16720728 10.1096/fj.05-5547fje

[69] G. Gambino , D. Gallo , A. Covelo , G. Ferraro , P. Sardo , and G. Giglia , “TRPV1 Channels in Nitric Oxide‐Mediated Signalling: Insight on Excitatory Transmission in Rat CA1 Pyramidal Neurons,” Free Radical Biology & Medicine 191 (2022): 128–136. 36029909 10.1016/j.freeradbiomed.2022.08.025

