# Supplementary material for: Restoration of Excitation‐Inhibition Balance and Improvement of Schizophrenia‐like Behavioral Deficits via Electroacupuncture
Source: CNS Neurosci Ther. 2026 Jun 10;32(6):e70971. doi: 10.1002/cns.70971 (PMC13250632; doi:10.1002/cns.70971)
Supplement: Supplementary file 1 — Figure S1: EA has no effect on time in center in open field test and latency to fall in rotarod test. (A) Scheme of experimental design. Breeding diagram for the generation of Erbb4CreER/+; nNosf/f mice (Erbb4‐nNos−/− mice). (B) Quantification of time in center from different groups in open field test. One‐way ANOVA with Bonferroni's multiple comparisons test, N = 12 per group; F = 0.3625, p = 0.7804. NS, not significant. (C) Quantification of latency to fall in rotarod test. Repeated two‐way ANOVA with Bonferroni's multiple comparisons test, N = 12 per group; F = 0.2441, p = 0.8651. Data are mean ± SEM. N indicates the number of biologically independent samples, mice per group. Figure S2: Acupuncture improves schizophrenia‐relevant behavioral deficits in Erbb4‐nNos−/− mice. (A‐B) Total distance (A) and time in center (B) from the different groups, including Erbb4‐nNos−/−, Erbb4‐nNos−/− + Sham and Erbb4‐nNos−/− + AC groups, in open field test. (A) One‐way ANOVA with Bonferroni's multiple comparisons test, N = 7–8 per group; F = 5.520, *p < 0.05. (B) One‐way ANOVA with Bonferroni's multiple comparisons test, N = 7–8 per group; F = 2.797, p = 0.0862. NS, not significant. (C‐D) Quantification of response to 70 dB (C) and percentage of PPI (D) in prepulse inhibition test. (C) One‐way ANOVA with Bonferroni's multiple comparisons test, N = 7–8 per group; F = 0.8013, p = 0.4633. NS, not significant. (D) Two‐way Repeated Measures ANOVA with Bonferroni's multiple comparisons test, N = 7–8 per group; F = 32.16, **p < 0.01. (E‐F) Percentage of spontaneous alternation (E) and the number of arm entries (F) in Y maze. (E) One‐way ANOVA with Bonferroni's multiple comparisons test, N = 7–8 per group; F = 5.291, *p < 0.01. (F) One‐way ANOVA with Bonferroni's multiple comparisons test, N = 7–8 per group; F = 0.0572, p = 0.9445. NS, not significant. Data are mean ± SEM. N indicates the number of biologically independent samples, mice per group. Figure S3: EA has little effect on basal [file CNS-32-e70971-s001.docx]

**Supplemental Materials**


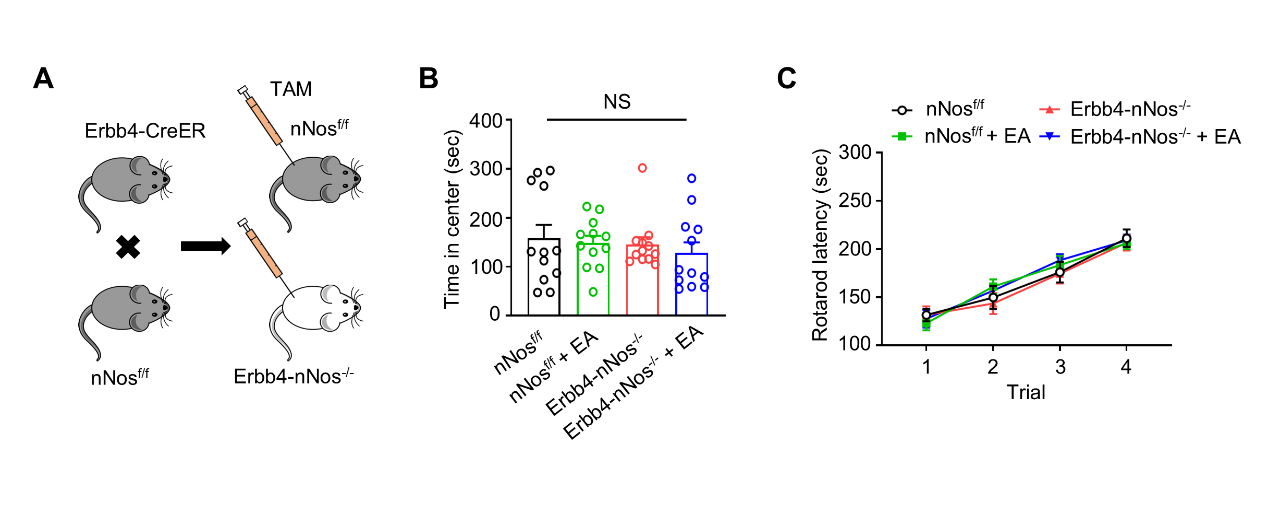


**Figure S1. EA has no effect on time in center in open field test and latency to fall in rotarod test. (A)** Scheme of experimental design. Breeding diagram for the generation of Erbb4^CreER/+^; nNos^f/f^ mice (Erbb4-nNos^-/-^ mice). **(B)** Quantification of time in center from different groups in open field test. One-way ANOVA with Bonferroni's multiple comparisons test, *N* = 12 per group; *F* = 0.3625, *P* = 0.7804. NS, not significant. **(C)** Quantification of latency to fall in rotarod test. Repeated two-way ANOVA with Bonferroni's multiple comparisons test, *N* = 12 per group; *F* = 0. 2441, *P* = 0.8651. Data are mean ± SEM. *N* indicates the number of biologically independent samples, mice per group.


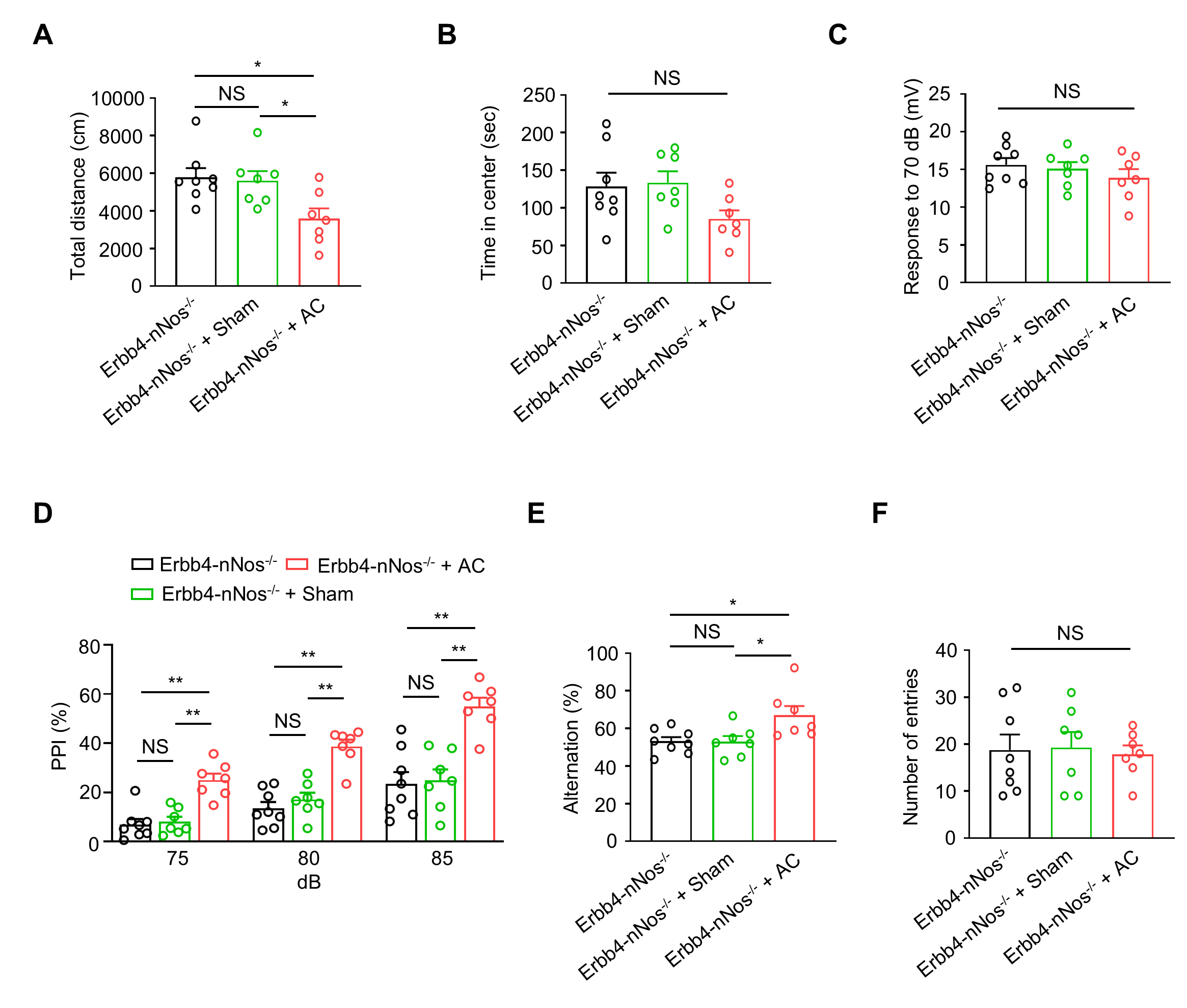


**Figure S2. Acupuncture improves schizophrenia-relevant behavioral deficits in Erbb4-nNos^-/-^ mice. (A-B)** Total distance **(A)** and time in center **(B)** from the different groups, including Erbb4-nNos^-/-^, Erbb4-nNos^-/-^ + Sham and Erbb4-nNos^-/-^ + AC groups, in open field test. **(A)** One-way ANOVA with Bonferroni's multiple comparisons test, *N* = 7-8 per group; *F* = 5.520, **P* < 0.05. **(B)** One-way ANOVA with Bonferroni's multiple comparisons test, *N* = 7-8 per group; *F* = 2.797, *P* = 0.0862. NS, not significant. **(C-D)** Quantification of response to 70 dB **(C)** and percentage of PPI **(D)** in prepulse inhibition test. **(C)** One-way ANOVA with Bonferroni's multiple comparisons test, *N* = 7-8 per group; *F* = 0.8013, *P* = 0.4633. NS, not significant. **(D)** Two-way Repeated Measures ANOVA with Bonferroni's multiple comparisons test, *N* = 7-8 per group; *F* = 32.16, ***P* < 0.01. **(E-F)** Percentage of spontaneous alternation **(E)** and the number of arm entries **(F)** in Y maze. **(E)** One-way ANOVA with Bonferroni's multiple comparisons test, *N* = 7-8 per group; *F* = 5.291, **P* < 0.01. **(F)** One-way ANOVA with Bonferroni's multiple comparisons test, *N* = 7-8 per group; *F* = 0.0572, *P* = 0.9445. NS, not significant. Data are mean ± SEM. *N* indicates the number of biologically independent samples, mice per group.


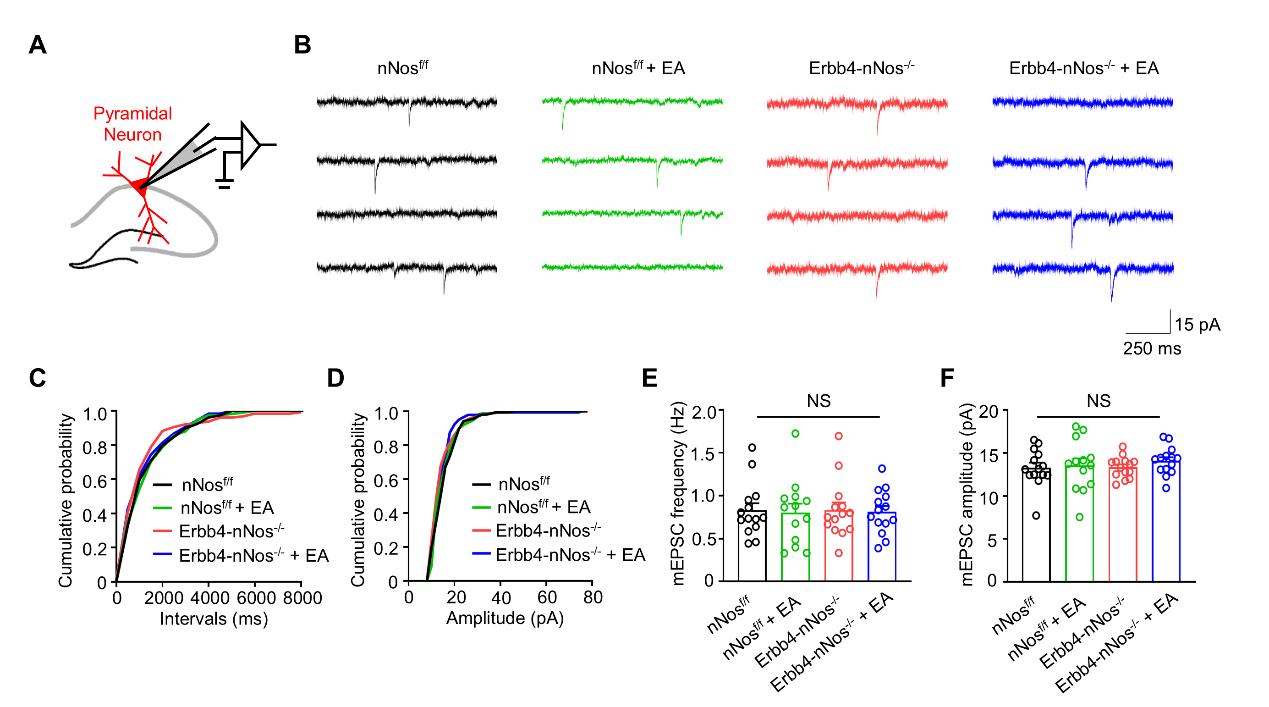


**Figure S3. EA has little effect on basal excitatory neurotransmission in hippocampus from Erbb4-nNos^-/-^ mice. (A)** Schematic representation of whole-cell recordings from pyramidal neurons in hippocampus. **(B)** Representative traces of mEPSCs in CA1 pyramidal neurons from four groups, including nNos^f/f^, nNos^f/f^ + EA, Erbb4-nNos^-/-^ and Erbb4-nNos^-/-^ + EA groups. Scale bar = 250 ms, 15 pA. **(C-D)** Cumulative plots of mEPSC interevent intervals **(C)** and amplitude **(D)**. **(E-F)** Quantification of mean values of mEPSC frequency **(E)** and amplitude **(F)**. **(E)** One-way ANOVA with Bonferroni's multiple comparisons test, *N* = 13/14 cells from 3 mice per group; *F* = 0.02459, *P* = 0.9947. NS, not significant. **(F)** One-way ANOVA with Bonferroni's multiple comparisons test, *N* = 13/14 cells from 3 mice per group; *F* = 0.4123, *P* = 0.7449. NS, not significant. Data are mean ± SEM. *N* indicates the number of biologically independent samples, mice per group.


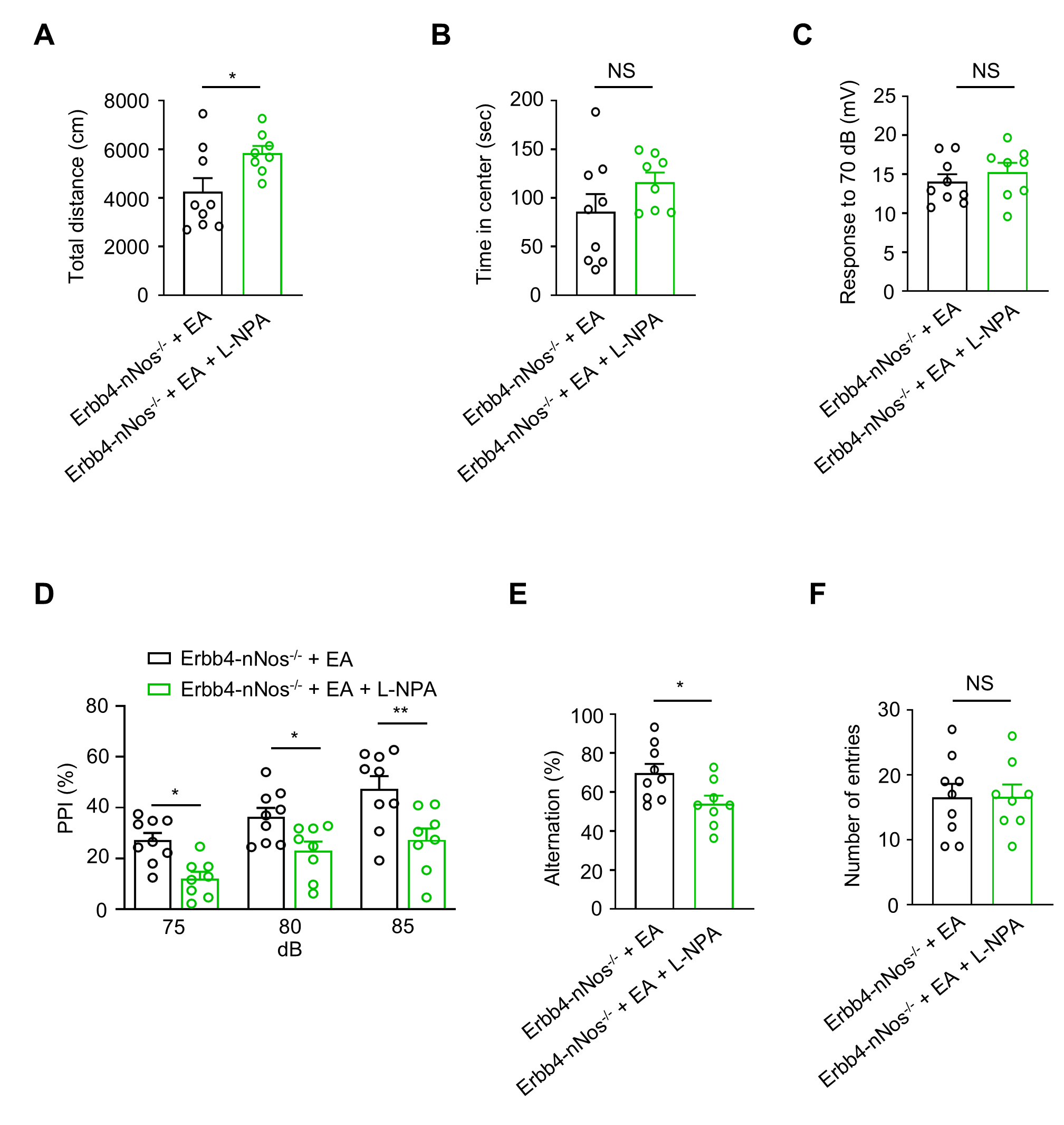


**Figure S4. L-NPA, a selective nNOS inhibitor, abolishes the therapeutic effect of EA in Erbb4-nNos^-/-^ mice. (A-B)** Total distance **(A)** and time in center **(B)** from two groups, including Erbb4-nNos^-/-^ + EA and Erbb4-nNos^-/-^ + EA + L-NPA groups, in open field test. **(A)** Unpaired t test, *N* = 8-9 per group; *P* = 0.0299, **P* < 0.05. **(B)** Unpaired t test, *N* = 8-9 per group; *P* = 0.1785. NS, not significant. **(C-D)** Quantification of response to 70 dB **(C)** and percentage of PPI **(D)** in prepulse inhibition test. **(C)** Unpaired t test, *N* = 8-9 per group; *P* = 0.4153. NS, not significant. **(D)** Two-way Repeated Measures ANOVA with Bonferroni's multiple comparisons test, *N* = 8-9 per group; *F* = 16.97, **P* < 0.05, ***P* < 0.01. **(E-F)** Percentage of spontaneous alternation **(E)** and the number of arm entries **(F)** in Y maze. **(E)** Unpaired t test, *N* = 8-9 per group; *P* = 0.0256, **P* < 0.01. **(F)** Unpaired t test, *N* = 8-9 per group; *F* = 0.1405, *P* = 0.9807. NS, not significant. Data are mean ± SEM. *N* indicates the number of biologically independent samples, mice per group.


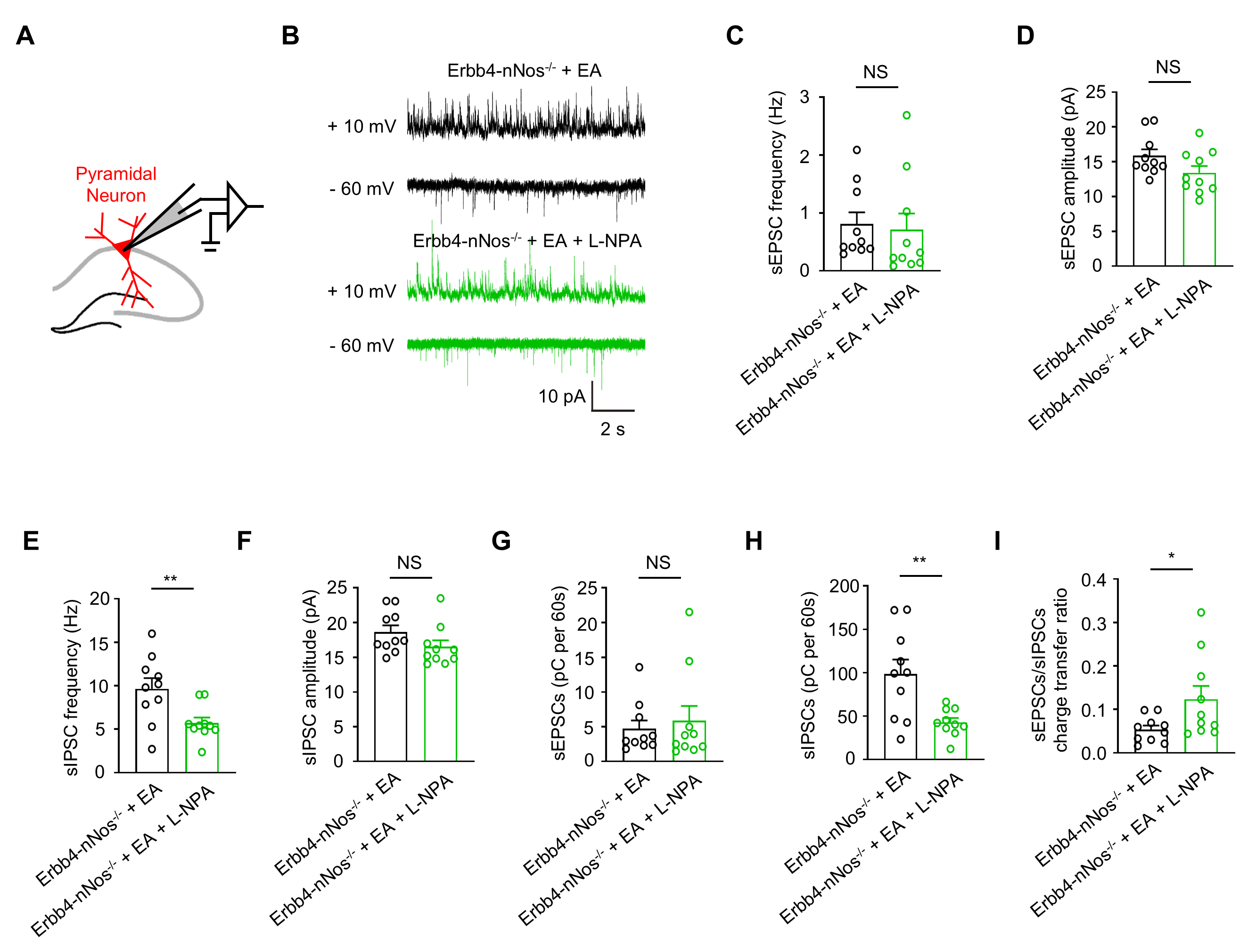


**Figure S5. L-NPA blocks the therapeutic effect of EA on E/I balance in Erbb4-nNos^-/-^ mice. (A)** Schematic representation of whole-cell recordings from pyramidal neurons in hippocampus. **(B)** Representative traces of sEPSCs (- 60 mV) and sIPSCs (+ 10 mV) in CA1 pyramidal neurons in two groups, including Erbb4-nNos^-/-^ + EA and Erbb4-nNos^-/-^ + EA + L-NPA groups. Scale bar = 2 s, 10 pA. **(C-D)** Quantification of mean values of sEPSC frequency **(C)** and amplitude **(D)**. **(C)** Unpaired t test, *N* = 10 cells from 3 mice per group; *P* = 0.7779. NS, not significant. **(D)** Unpaired t test, *N* = 10 cells from 3 mice per group; *P* = 0.0850. NS, not significant. **(E-F)** Quantification of mean values of sIPSC frequency **(E)** and amplitude **(F)**. **(E)** Unpaired t test, *N* = 10 cells from 3 mice per group; *P* = 0.0098. ***P* < 0.01. **(F)** Unpaired t test, *N* = 10 cells from 3 mice per group; *P* = 0.1241. NS, not significant. **(G)** Quantification of sEPSCs from four groups. Unpaired t test, *N* = 10 cells from 3 mice per group; *P* = 0.6442. NS, not significant. **(H)** Quantification of sIPSCs from four groups. Unpaired t test, *N* = 10 cells from 3 mice per group; *P* = 0.0046. ***P* < 0.01. **(I)** Quantification of sEPSC/sIPSC charge transfer ratios. Unpaired t test, *N* = 10 cells from 3 mice per group; *P* = 0.0417. **P* < 0.05. Data are mean ± SEM. *N* indicates the number of biologically independent samples, mice per group.
